# Assembly and turnover of neurofilaments in growing axonal neurites

**DOI:** 10.1242/bio.028795

**Published:** 2017-11-20

**Authors:** Edward F. Boumil, Rishel Vohnoutka, Sangmook Lee, Harish Pant, Thomas B. Shea

**Affiliations:** 1 Laboratory for Neuroscience, University of Massachusetts Lowell, Lowell, MA 01854, USA; 2 Cytoskeletal Protein Regulation Section, National Institute of Neurological Disorders and Stroke, National Institutes of Health, Bethesda, MD 20892 , USA

**Keywords:** Neurofilament, Cytoskeleton, Axon, Axogenesis, Axonal transport

## Abstract

Neurofilaments (NFs) are thought to provide stability to the axon. We examined NF dynamics within axonal neurites of NB2a/d1 neuroblastoma by transient transfection with green fluorescent protein-tagged NF-heavy (GFP-H) under the control of a tetracycline-inducible promoter. Immunofluorescent and biochemical analyses demonstrated that GFP-H expressed early during neurite outgrowth associated with a population of centrally-situated, highly-phosphorylated crosslinked NFs along the length of axonal neurites (‘bundled NFs’). By contrast, GFP-H expressed after considerable neurite outgrowth displayed markedly reduced association with bundled NFs and was instead more evenly distributed throughout the axon. This differential localization was maintained for up to 2 weeks in culture. Once considerable neurite outgrowth had progressed, GFP that had previously associated with the NF bundle during early expression was irreversibly depleted by photobleaching. Cessation of expression allowed monitoring of NF turnover. GFP-H associated bundled NFs underwent slower decay than GFP-H associated with surrounding, less-phosphorylated NFs. Notably, GFP associated with bundled NFs underwent similar decay rates within the core and edges of this bundle. These results are consistent with previous demonstration of a resident NF population within axonal neurites, but suggest that this population is more dynamic than previously considered.

## Abstract (precis)

**Summary:** Immunofluorescent and radiolabel analyses demonstrate that neurofilaments establish a resident population within growing axonal neurites that undergoes exchange with a surrounding, transporting pool.

## INTRODUCTION

Neurofilaments (NFs) are heteropolymers composed of proteins, termed NF-light (NF-L), NF-medium (NF-M) and NF-heavy (NF-H) with respect to their molecular masses (Liem et al., 1978) along alpha-internexin and peripherin in central and peripheral neurons, respectively (Yuan et al., 2006a, 2012). C-terminal tail domains of NF subunits project laterally from the filament core (Hirokawa et al., 1984; Hisanaga and Hirokawa, 1988), and are the substrate for numerous kinases and phosphatases (Lee et al., 2014). NF subunits have C-terminal tails of differing lengths, with NF-H having the largest tail domain. NFs that have undergone extensive C-terminal tail-domain phosphorylation are normally segregated within axons (Pant and Veeranna, 1995). Phosphorylation of the NF-H C-terminal domain protects NFs from proteolysis (Goldstein et al., 1987; Pant, 1988; Greenwood et al., 1993; Rao et al., 2012) and promotes divalent cation-mediated associations between NFs and with other cytoskeletal elements, which generates a cytoskeletal lattice along the length of axons (Gotow and Tanaka, 1994; Gotow et al., 1994; Yabe et al., 2001a,b; Kushkuley et al., 2009). This ‘resident’ or stationary population is thought to be formed and maintained by continuous exchange with more rapidly transporting NFs/NF subunits; a population of highly-phosphorylated NFs provides stability to axons, while a population of more relentlessly transporting, poorly-phosphorylated NFs repairs and regenerates this stationary cytoskeleton (Nixon and Logvinenko, 1986; Rao et al., 2012; Lee and Shea, 2012).

The degree of C-terminal phosphorylation is inversely proportional to the rate of NF transport. The front of the transporting wave of newly-synthesized NFs is enriched in hypophosphorylated NFs and undergoes transport along the length of axons within days, while NFs displaying C-terminal phosphorylation persist along axons for months and these persisting NFs are depleted uniformly (Nixon and Logvinenko, 1986; Lewis and Nixon, 1988; Jung and Shea, 1999; Jung et al., 2000a,b, 2004). Notably, depletion of the NF-H C-terminal tail does not increase the transport rate of the fastest-moving NFs but prevents the slowing of NFs that otherwise accompanies C-terminal phosphorylation (Rao et al., 2012; Yuan et al., 2006b). Conversely, knockout of the entire NF-H fosters continued association of NFs with their anterograde transport protein kinesin (Jung et al., 2006). These studies collectively suggest that NF C-terminal phosphorylation does not directly impede axonal transport, but does so indirectly by fostering NF-NF associations that compete with transport and establish a more ‘resident’ population that likely undergoes continuous exchange with more rapidly transporting NFs (Shea and Lee, 2011).

Prior studies using the reductionist approach of expression of NF subunits conjugated to green fluorescent protein (GFP) in cultured neurons and neuroblastoma have allowed monitoring of NF transport and incorporation into the cytoskeleton of axons and axonal neurites (Yabe et al., 1999, 2001a,b; Yuan et al., 2009; Wang et al., 2000; Roy et al., 2000). These studies have demonstrated that, while NFs undergo transport at rapid speeds consistent with their known motor proteins kinesin, dynein and myosin (Yabe et al., 1999; Jung et al., 2004; Alami et al., 2009; Shah et al., 2000; Motil et al., 2006; Uchida et al., 2009), only a relatively small percentage of NFs are associated with their motors at any time (Yabe et al., 1999, 2000), and only for relatively short intervals (Brown et al., 2005; Wang et al., 2000; Wang and Brown, 2001). These studies have further demonstrated that NFs can be harvested separated as two distinct two populations: a centrally-situated population of closely-opposed NFs that display a high degree of C-terminal phosphorylation with a consequential high-degree of divalent cation-mediated phospho-phospho associations, and a ‘surrounding’ population of more widely dispersed, more rapidly transporting NFs that display fewer phospho-epitopes (Yabe et al., 2001a,b; Kushkuley et al., 2009). Formation of centrally-situated NF ‘bundles’ is temporally and spatially restricted to accumulation of a critical level of NFs within axons/axonal neurites (Yuan et al., 2009; Yabe et al., 2001a,b; Kushkuley et al., 2009). While there is a likely continuum between these populations, they were nevertheless experimentally separable by differential centrifugation of cultures as well as sciatic nerve. In addition, exchange of subunits between these populations has been observed within cultured neurons/neuroblastoma (Yuan et al., 2009; Yabe et al., 2001a,b; Trivedi et al., 2007). Following isolation from cultures, spinal cord and sciatic nerve, these populations could be inter-converted (i.e. bundled NFs could be dissociated and individual NFs could undergo phospho-mediated interactions) by manipulation of kinases, phosphatases and calcium levels (Kushkuley et al., 2009; Lee et al., 2014). As in axons *in situ*, this segregation is regulated by differential association of NFs with motor proteins coupled with phospho-mediated NF-NF associations that foster at least transient withdrawal of NFs from the transporting pool (Shea and Lee, 2011; Lee and Shea, 2012). Bundled NFs have been suggested to correspond to the resident NFs observed following radiolabeling *in situ* (Yabe et al., 2001a,b; Yuan et al., 2009, 2012; Shea et al., 2009; Lee and Shea, 2012).

NF dynamics, including formation of NF bundles, have been extensively studied in NB2a/d1 cells using GFP-tagged NF subunits (Ackerley et al., 2003; Boumil et al., 2015; Chan et al., 2003, 2004, 2005; Dubey et al., 2007; Kushkuley et al., 2009; Lee and Shea, 2014; Lee et al., 2011a,b, 2014; Moran et al., 2005; Motil et al., 2006, 2007; Sunil et al., 2012; Vohnoutka et al., 2017; Yabe et al., 1999, 2001a,b). Complete elucidation of the nature and extent of exchange of NF subunits between resident bundled NFs and more labile, rapidly-transporting individual NFs has been hindered by relative quick saturation of the entire cytoskeleton with GFP-tagged subunits (e.g. within 24-36 h) due to their continuous expression (Yabe et al., 2001a,b). In efforts to surmount this problem, we transiently expressed GFP-H under the control of a tetracycline-inducible promoter system, which has previously been shown to facilitate monitoring of NF-H turnover (Szebenyi et al., 2002). As described herein, expression of GFP-H at different stages of axonal neurite outgrowth allowed us to demonstrate continuous NF subunit exchange between rapidly-transporting and resident populations.

## RESULTS

### The tetracycline-inducible system produces tight regulation of gene expression

**Fig. 1.**
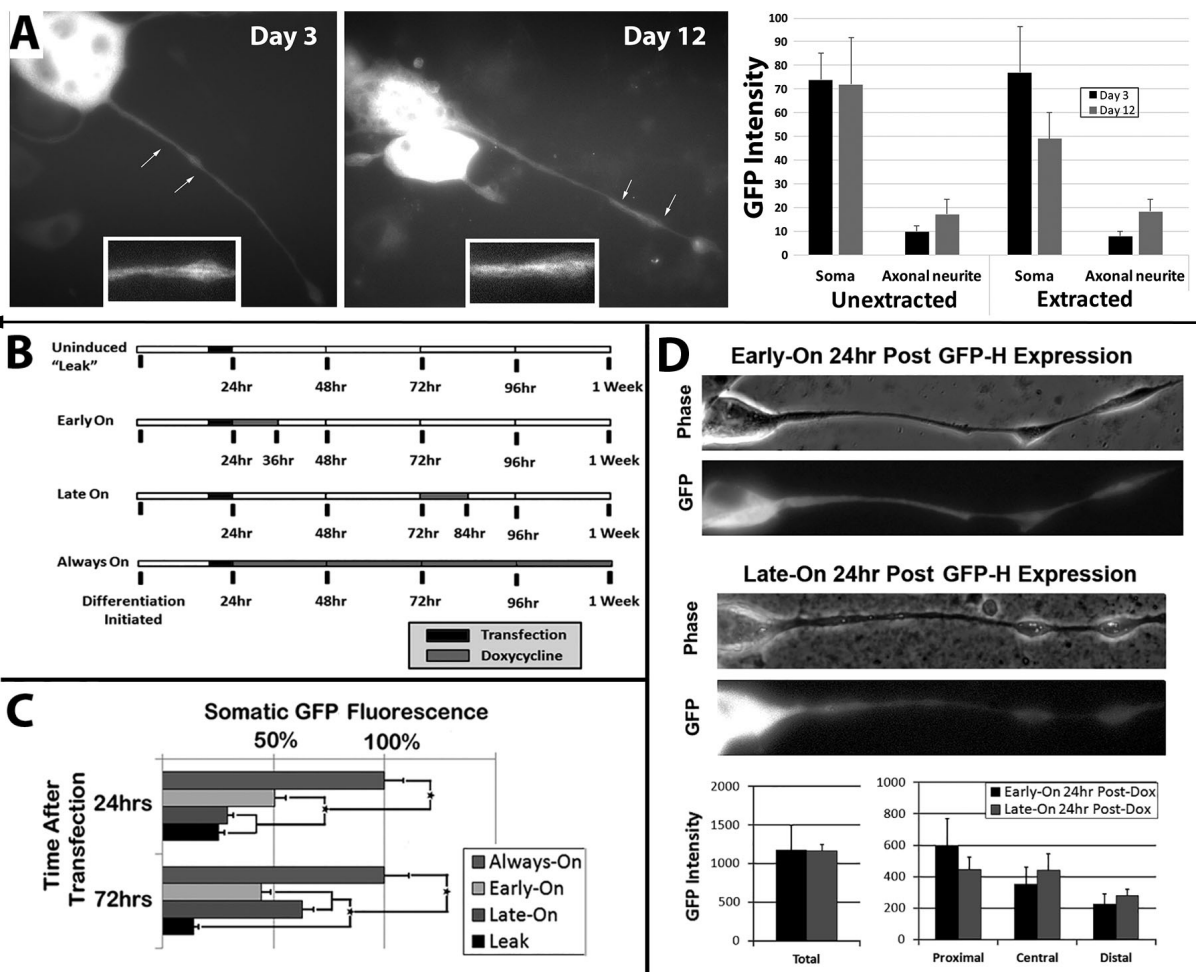
**Experimental outline and validation of tet-inducible expression system.** (A) Representative NB2a/d1 cells at day 3 and 12 after initiation of dbcAMP-induced differentiation, transiently transfected with GFP-H on day 2 after initiation of differentiation. Insets show higher-magnification views of regions of axonal neurites indicated by arrows. Note association of GFP with filamentous structures along the length of axonal neurites along with saturation of the soma. (B) Timeline of induction and cessation of GFP-H expression under control of the tet-inducible promoter by addition of doxycycline (dox) for 12 h after 24 h or 72 h of differentiation (‘Early-On’ and ‘Late-On’, respectively). Additional cultures did not receive dox (‘Leak’) or received dox for 1 week (‘Always-On’). (C) Quantification of GFP under the conditions described in panel B at 24 and 72 h after transfection (*n*=total of >1000 cells from three separate experiments; >65 cells/condition/time point). *P<0.01. (D) Quantification of GFP within total axonal neurites, or three segments of equivalent length (proximal, central and distal) under Early-On and Late-On conditions as described in B at 24 and 72 h after transfection (*n*=39 total cells).

NB2a/d1 cells were transiently transfected with GFP-H 48 h after the initiation of differentiation (Fig. 1A). As in prior studies, GFP-H was associated with filamentous structures along the length of axonal neurites within 24 h, and was retained for many days (Fig. 1A). However, continuous expression for as little as 24 h saturates both the soma and neurites, which leaves only a short window for observation of the dynamics of transport and cytoskeletal incorporation (Yabe et al., 2001a,b), and precludes most analyses of NF turnover following this brief interval. To provide control over expression levels, differentiating NB2a/d1 cells were transiently transfected with GFP-H under the control of a tet-inducible promoter, after which expression was induced for 12 h after 24 h of differentiation (which is prior to establishment of a resident NF population within neurites) and in other cells after 72 h of differentiation (which is after establishment of a resident NF population). These conditions were termed ‘Early-On’ and ‘Late-On’, respectively (Fig. 1B). Expression was induced after 24 h of differentiation and allowed to continue for 1 week in additional cultures (‘Always-On’). Always-On cultures displayed significantly higher levels of GFP within 24 h than those that were transfected but in which expression was not induced (termed ‘Leak’ cultures due to minor promoter expression in the absence of induction), and the increased levels in Always-On cultures were as retained at 72 h after transfection (Fig. 1C). At 24 h after transfection, Early-On cultures displayed significantly more somatic GFP than Leak cultures, yet significantly less than Always-On cultures. Levels in soma of Early-On cultures had declined slightly by 72 h after transfection.

At 24 h after transfection, Late-On cultures displayed GFP levels identical to those of Leak cultures, consistent with expression not yet having been induced. By 72 h after transfection, soma of Late-On cultures displayed more GFP than that of Early-On cultures, but less than that of Always-On cultures. Leak cultures did not change between 24 and 72 h after transfection (Fig. 1C). These findings support regulation of gene expression using this system, including the ability to create a 12 h ‘pulse’ of expression that can facilitate tracking of NF dynamics by avoiding cytoskeletal saturation characteristic of continuous expression.

### Distribution of GFP following differential expression

**Fig. 2.**
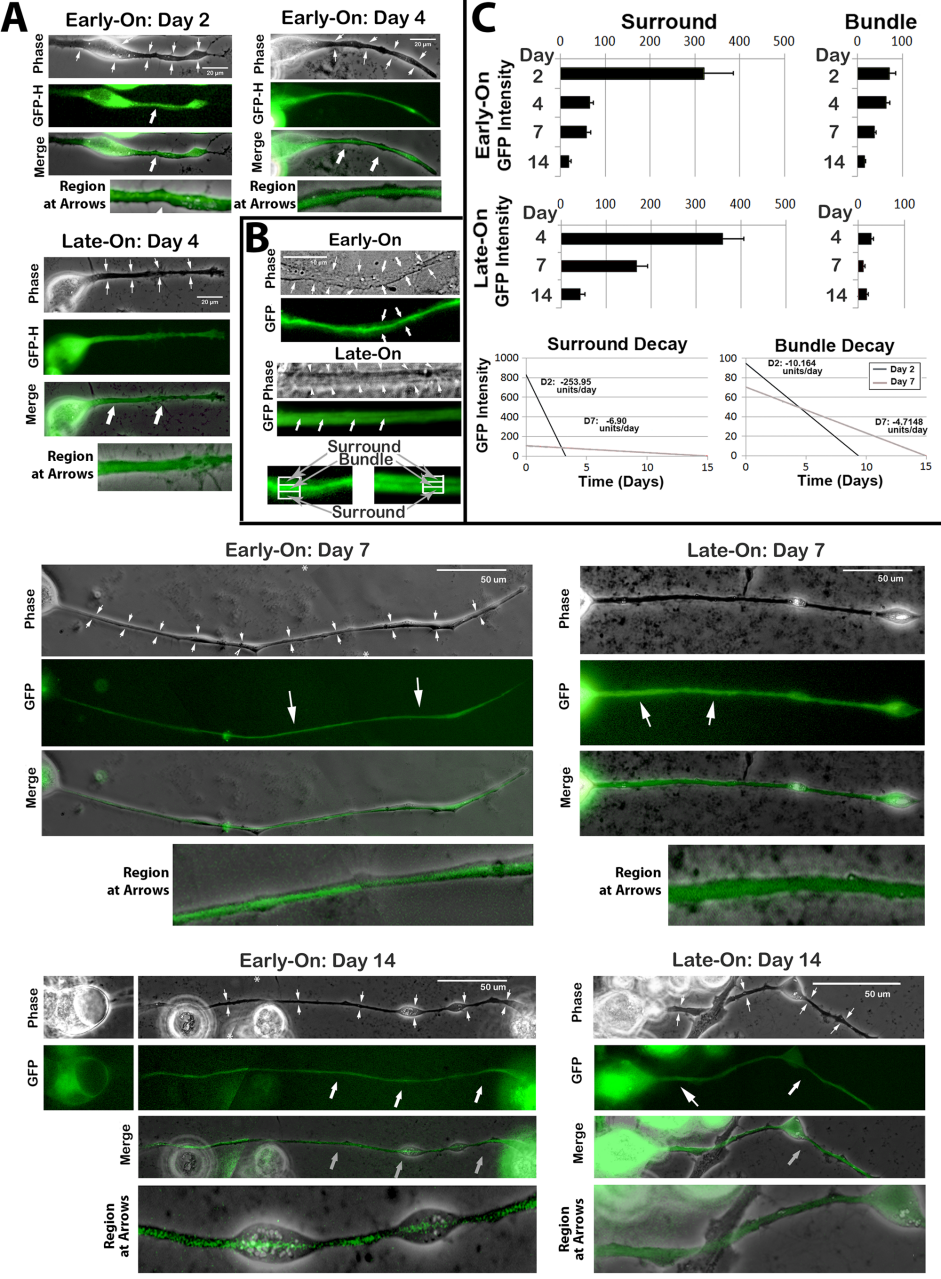
**Differential distribution of GFP under Early-On and Late-On conditions.** (A) Representative phase-contrast and corresponding epifluorescent images of Early-On and Late-On cells at the indicated day after induction of differentiation. Arrows in epifluorescent images denote the distribution of GFP; arrows in corresponding phase-contrast images denote the diameter of the axonal neurite. Larger arrows in the merged images also denote the region from which the higher-magnification image was derived. By days 7-14, axonal neurites were often too long for single micrographs at 100×. The ‘Early-On’ axonal neurite micrographs presented for these times in Fig. 2 are composites of two micrographs. Asterisks in the respective phase-contrast images indicate where composite images were merged. (B) Higher-magnification images from representative cells at day 4 after induction of differentiation. Note differential distribution of GFP within the centrally-situated NF bundle and was instead confined to the area surrounding the bundle (‘surround’). Arrows in the phase-contrast image denote the diameter of the axonal neurite. (C) Upper graphs present quantification of distribution of GFP in Early-On and Late-On axonal neurites from multiple cells at the indicated day after induction of differentiation. Lower graphs present the rate of decline of GFP from the surround and from the bundle at days 2 and 7 after induction of differentiation (*n*=total 96 cells from four separate experiments; *P*<0.01 for Early-On day 4 versus Late-On day 4 at both 24 and 48 h).

Overall levels of GFP were quantified within axonal neurites of Early-On and Late-On cultures 24 h after expression under each condition (Fig. 1D). Total neuritic GFP and GFP distribution along the length of axonal neurites were statistically identical within Early-On and Late-On cultures 24 h after their respective expression times. These findings indicate that NF transport was not perturbed by differential timing of GFP-H expression. However, GFP was differentially associated with centrally-situated longitudinal NF bundles within Early-On versus Late-On cultures. GFP localized within NF bundles within 24 h after expression in Early-On cultures (day 2 of differentiation) and was retained within bundles for 2 weeks (the longest time examined) (Fig. 2A). By contrast, GFP was not localized within NF bundles 24 h after expression in Late-On cultures (day 4 of differentiation), but was instead more diffuse throughout axonal neurites. Higher-magnification images revealed that GFP was apparently restricted from association with the centrally-situated NF bundle and was instead confined to the area surrounding the bundle (henceforth termed the ‘surround’) at this interval (Fig. 2B). At later times, however, (e.g. days 7-14), GFP was apparently associated with the centrally-situated NF bundle in Late-On cultures (Fig. 2A). We therefore quantified the relative distribution of GFP within axonal neurites at multiple times following expression. Early-On and Late-On axons displayed statistically identical levels of GFP within the surround at 24 h post-induction of expression (day 2 and day 4 after differentiation, respectively (Fig. 2C); however, >2.5-fold more GFP was associated with NF bundles 24 h after expression in Early-On versus Late-On cultures at this time. Significantly higher GFP levels were associated with NF bundles in Early-On versus Late-On cultures for up to and including 7 days in culture. However, levels within bundles of Early-On cells declined significantly over time such that, by day 14 in culture, statistically-identical GFP was associated with NF bundles in Early-On and Late-On cells.

These analyses also demonstrated differential depletion of GFP within the surround versus the NF bundle. The surround initially contained much higher levels of GFP than that of the NF bundle in both Early-On and Late-On cells; however, for both of these conditions, GFP was depleted from the surround more rapidly that that associated with the NF bundle (Fig. 2C). This was further substantiated by comparison of the half-life of GFP in the surround and bundle in Early-On cells. The half-life of GFP in the surround was 0.9±0.04 days between days 2-4 in culture, and increased to 5.7±0.5 days between days 4-14 in culture. By contrast, the half-life of GFP in the bundle was 5.5±0.1 days between the entire span of days 2-14 in culture. The rate of GFP decline in the surround was 25× that in the bundle at day 2 in culture. By day 7, the rate of GFP decline in the surround had decreased to only 1.5× of that of GFP within the bundle (Fig. 2C).

**Fig. 3.**
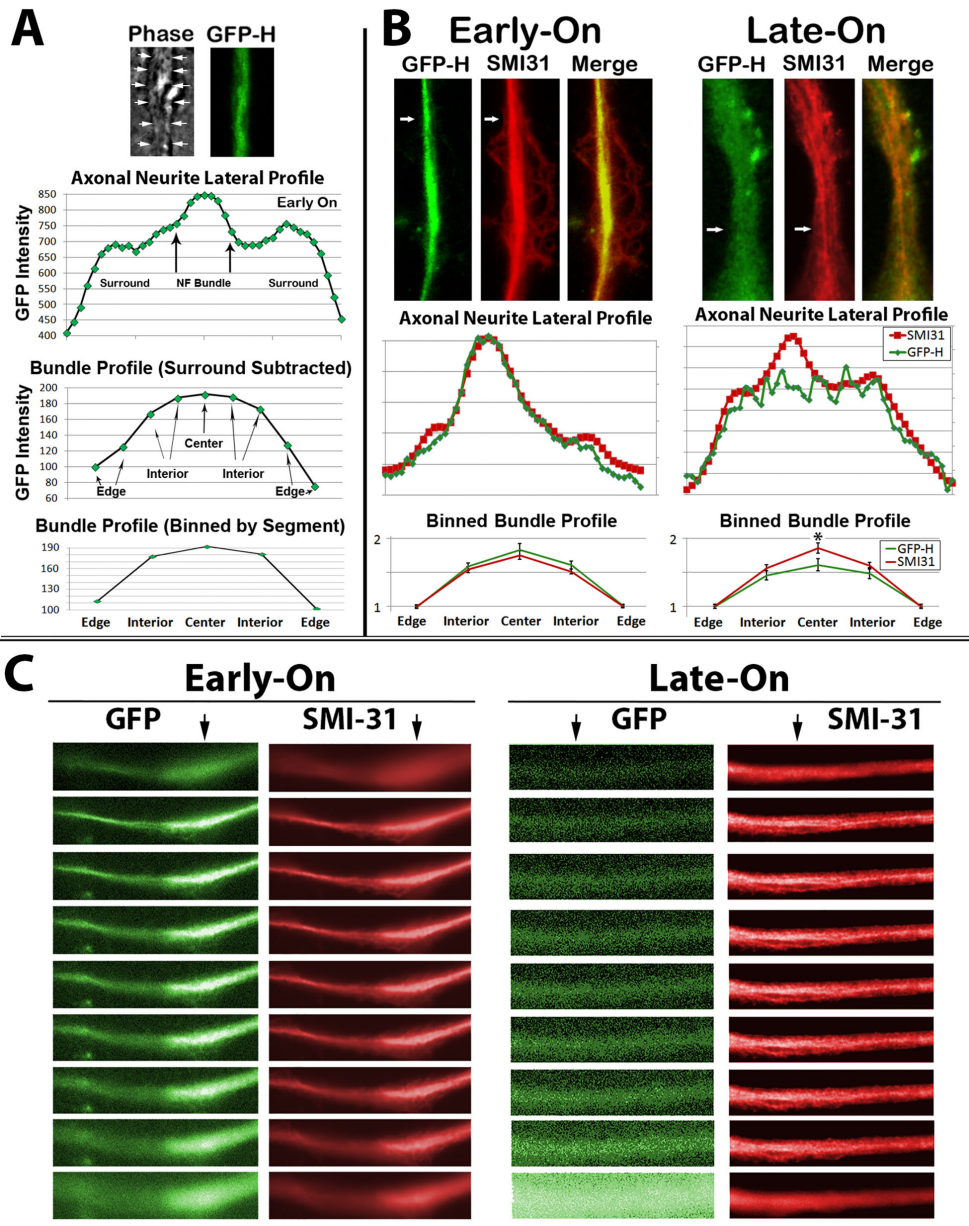
**Differential distribution of GFP under Early-On and Late-On conditions.** (A) Representative phase-contrast and epifluorescent image of the central region of an axonal neurite. Arrows in the phase-contrast image denote the diameter of the axonal neurite. The accompanying graphs present the relative distribution of GFP across the lateral profile (axonal neurite lateral profile), the distribution of GFP across the bundle (bundle profile) and the distribution of GFP within the bundle following binning as described in the Materials and Methods to normalize distribution among multiple profiles. (B) Representative images and quantification of the distribution of GFP and total phospho-H as revealed by SMI-31 immunoreactivity in central segments of axonal neurites as described in A. Arrows denote the region from which respective lateral profiles were generated. Lower graphs present binned distribution from bundles from 53 of such cells. (C) Representative deconvoluted Z-stacks depicting the distribution of GFP and SMI-31 in Early-On and Late-On axonal neurites after 24 h of respective GFP-H expression.

It has been considered that the central-most NFs within a bundle may display the longest residence time within bundles, while those on the edge of the bundle may undergo exchange with surrounding, non-bundled NFs (Yabe et al., 2001a,b; Shea and Yabe, 2000; Chan et al., 2005). To determine whether or not this is the case, we selected regions of axonal neurites mid-way between the hillock and growth cone, and compared the distribution of GFP and phospho-NF immunoreactivity laterally across axonal neurites (Fig. 3A). The distribution of total phospho-NFs within lateral axonal profiles as revealed by SMI-31 analysis was identical in Early-On and Late-On axonal neurites (Fig. 3B). However, while the distribution of SMI-31 immunoreactivity in Late-On cells highlighted the NF bundle, GFP did not colocalize within the bundle within Late-On axonal neurites (Fig. 3B). We then compared the lateral distribution of GFP within bundles themselves. To normalize values from multiple axonal neurites, profiles of bundles were binned into five lateral segments (Fig. 3A). The distribution of total phospho-NFs as revealed by SMI-31 analysis was identical in Early-On and Late-On bundles (Fig. 3B). However, while GFP distribution within Early-On bundles was identical to that of total phospho-NFs, GFP within the central segment of Late-On bundles was statistically lower than that of total phospho-NFs (Fig. 3B).

Confocal analyses further corroborated differential distribution of GFP depending upon the timing of expression. A deconvoluted Z-stack of axonal neurites images from Early-On and Late-On cells after 24 h of respective GFP-H expression demonstrated that GFP in Early-On axonal neurites co-localized with the centrally-situated NF bundle as visualized by immunofluorescent analyses with the phospho-dependent NF antibody SMI-31, while GFP was instead evenly dispersed throughout Late-On axonal neurites and did not colocalize with the NF bundle (Fig. 3C).

### Biochemical analyses of GFP-H distribution following differential expression

**Fig. 4.**
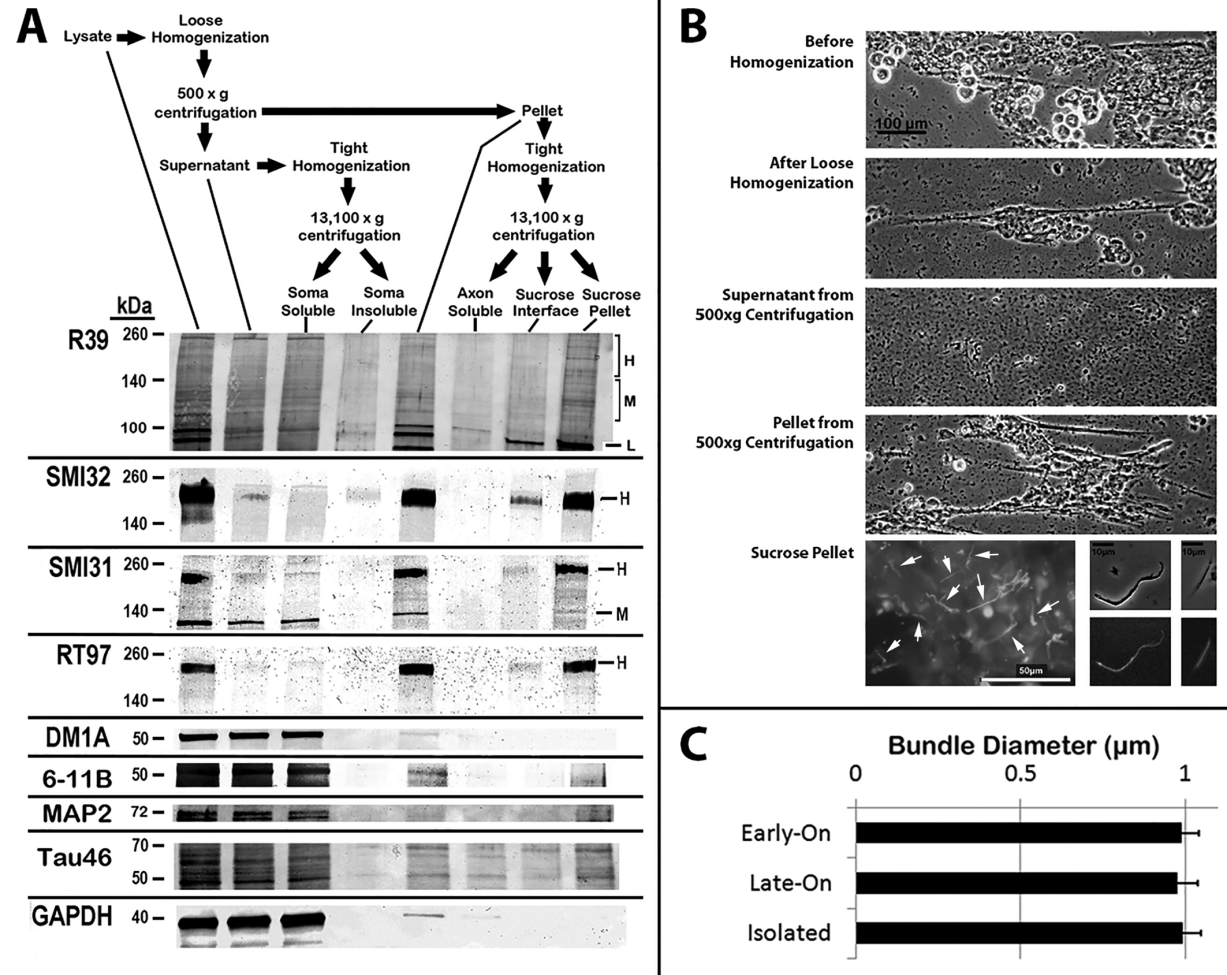
**Fractionation and differential centrifugation methodology.** (A) Fractionation and differential centrifugation protocol to separate axonal neurites from soma, Triton-soluble material and Triton-insoluble cytoskeletons, and bundles from individual NFs from cytoskeletons of axonal neurites as described in the Materials and Methods (Shea et al., 1993). The accompanying nitrocellulose replicas present distribution of total NFs (R39), non-phosphorylated NF-H and M (SMI-32), phosphorylated NF-H and M (SMI-31, RT97), total (DM1A) and acetylated (6-11B) tubulin, MAP2, tau (Tau46) and GAPDH as indicated. (B) Phase-contrast and epifluorescent images of fractions during the above procedure as indicated. Arrows denote NF bundles recovered in the sucrose pellet; not all bundles are indicated. The accompanying graph presents quantification of the diameter of bundles before and following isolation as indicated.

**Fig. 5.**
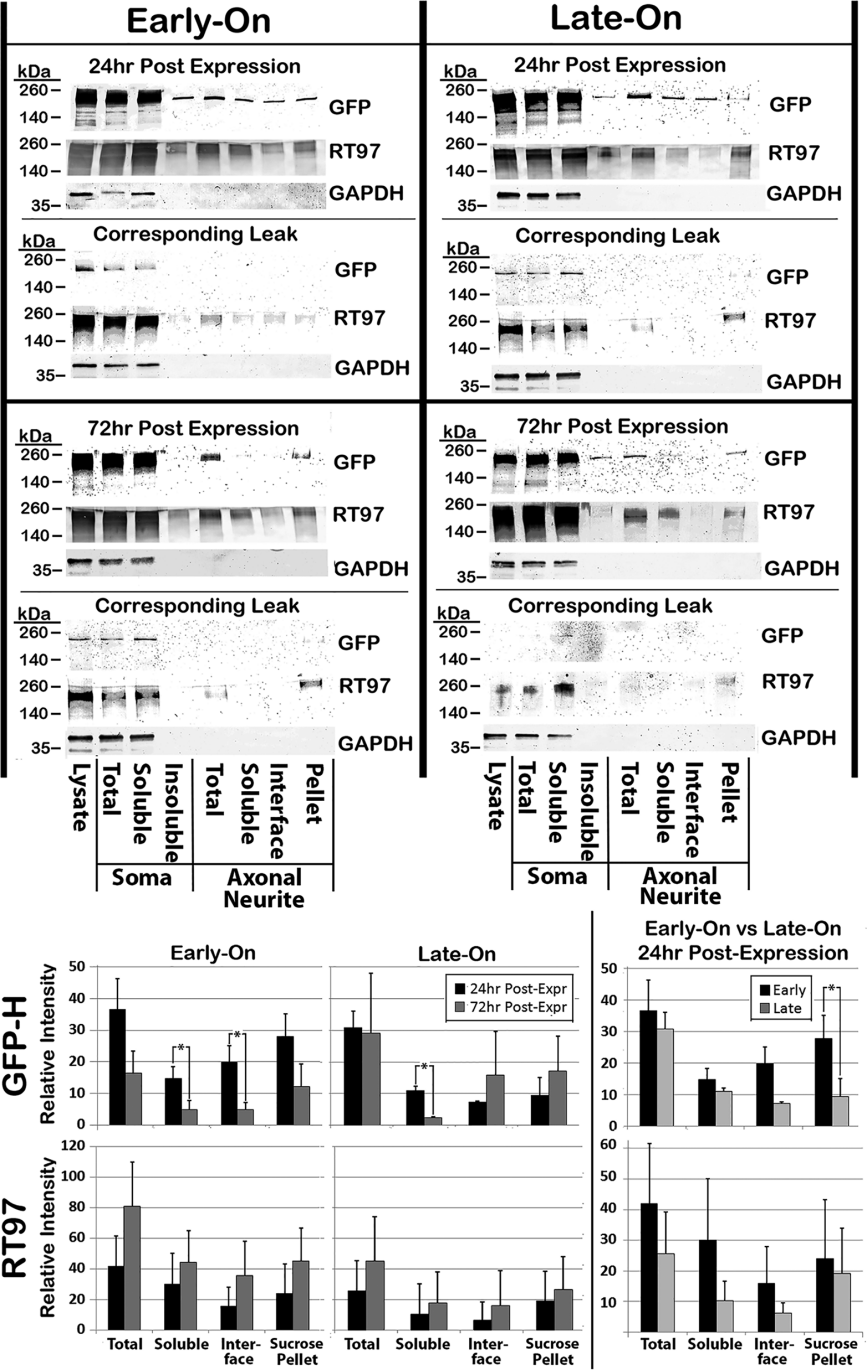
**Biochemical analyses of GFP-H distribution following differential expression.** Nitrocellulose replicas probed as indicated of fractions from Early-On and Late-On cells harvested at 24 and 72 h after expression as indicated, along with uninduced cells harvested at the same time (‘Corresponding Leak’). The accompanying graphs present quantification of the distribution of GFP-H and phospho-NF (RT97) among fractions from three experiments, and the ratio of distribution in Early-On versus Late-On cells as indicated (*P<0.05).

Biochemical analyses corroborated the differential distribution of GFP as observed in immunofluorescent analyses. Cells were subjected to a differential centrifugation protocol that separates axonal neurites from soma, followed by separation of Triton-soluble material and Triton-insoluble cytoskeletons, and finally, separation of NF bundles from individual NFs derived from cytoskeletons of axonal neurites (Fig. 4) (Shea et al., 1993). Similar levels of GFP-H were recovered within total lysates within axonal neurite cytoskeletons from both Early-On and Late-On cells at 24 h after respective induction of GFP-H expression (Fig. 5). Similar levels of GFP-H were also recovered within axonal neurite cytoskeletons from both Early-On and Late-On cells at 24 h after respective induction of GFP-H expression. These findings corroborate similar expression of GFP-H and transport into axonal neurites under both conditions (Fig. 1). Axonal neurite cytoskeletons were then further fractionated by sedimentation over a sucrose cushion as described in Fig. 4. Previous analyses demonstrated that only NF bundles sedimented through this cushion, while individual NFs instead collected at the interface between the buffer and this cushion (Shea et al., 1993). Significantly more GFP-H was recovered within the sucrose pellet in Early-On versus Late-On cells, while more GFP-H was recovered at the sucrose interface in Late-On versus Early-On cells (Fig. 5). While GFP-H within the Triton-soluble axonal neurite fraction and that recovered at the sucrose interface declined by 72 h, GFP-H within the sucrose pellet did not decline by 72 h. GFP-H within the Triton-soluble axonal neurite fraction from Late-On cells declined by 72 h, but levels recovered at the sucrose interface or sucrose pellet did not change (Fig. 5).

### Manipulation of Early-On and Late-On cultures supports differential incorporation of GFP-H within bundles

**Fig. 6.**
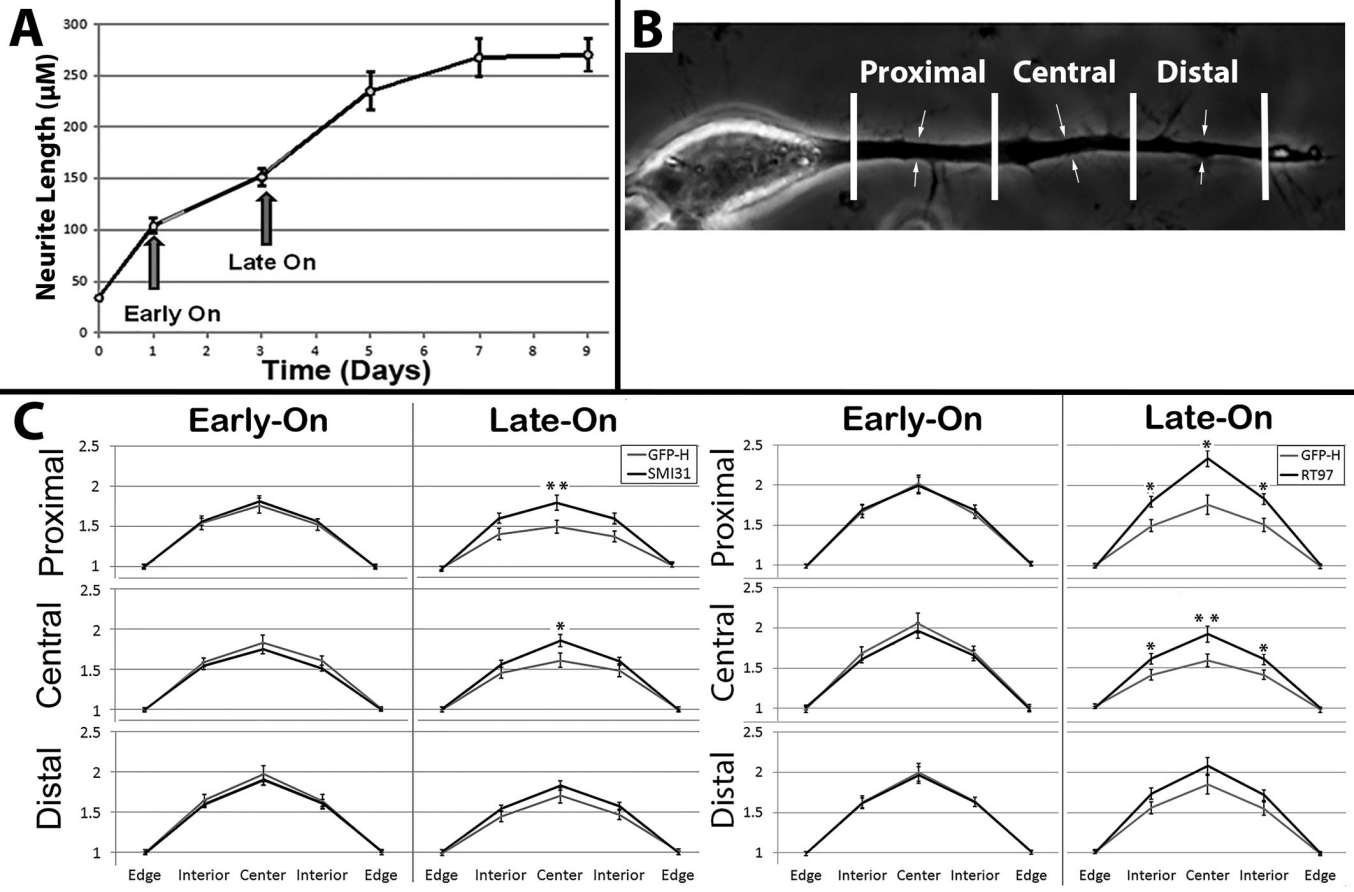
**Distribution of GFP within bundles along the length of axonal neurites.** (A) Quantification of axonal neurite length during dbcAMP-induced outgrowth. The timing of induction of GFP-H expression under Early-On and Late-On conditions is indicated. (B) Phase-contrast image of representative axonal neurite from day 7 after induction of differentiation, depicting segmentation of axonal neurites, excluding the hillock and growth cone, into three equivalent segments (proximal, central and distal as indicated). Arrows denote the diameter of the axonal neurite. (C) Quantification of distribution of GFP and phospho-H (RT97 in lateral profiles of bundles generated as described for Fig. 3, for proximal, central and distal segments as described in B from a total of 198 cells. *P<0.05, **P<0.01.

The above findings collectively support the hypothesis that the centrally-situated NF bundle represents a resident NF population that undergoes slower turnover than surrounding individual NFs (Yabe et al., 2001a,b; Yuan et al., 2009). One interpretation of our findings is that GFP-H is more readily incorporated into NF bundles in Early-On cells since Early-On cells formed bundles after/during GFP-H expression, and conversely, GFP-H was relatively restricted from incorporation into bundles in Late-On cells because these cells had already formed bundles before GFP-H was expressed. We noted, however, that axonal neurites continued elongating during times corresponding to Late-On expression (Fig. 6A). In the above analyses (Fig. 3), we selected central regions along the length of growing axonal neurites. If the above line of reasoning is correct, we considered that GFP-H should incorporate into NF bundles within the distal-most regions of axonal neurites during Late-On conditions, since, unlike central neurite regions, NF bundles in distal-most regions would form after GFP-H was expressed in Late-On cells. We therefore conducted lateral profile analyses from proximal, central and distal axonal neurite segments 24 h after expression of GFP-H in Early-On and Late-On cells as conducted for central segments alone in Fig. 3 (Fig. 6B). Total phospho-NFs within bundles as quantified by the phospho-dependent antibodies SMI31 and RT97 did not differ between Early-On and Late-On cells in any segment (Fig. 6C). GFP distribution within Early-On bundles was identical to that of both phospho-H antibodies within all three segments. GFP distribution within Late-On bundles was statistically reduced compared to both antibodies in proximal and central segments, but, conversely, was statistically identical to that of both antibodies in distal segments (Fig. 6C).

**Fig. 7.**
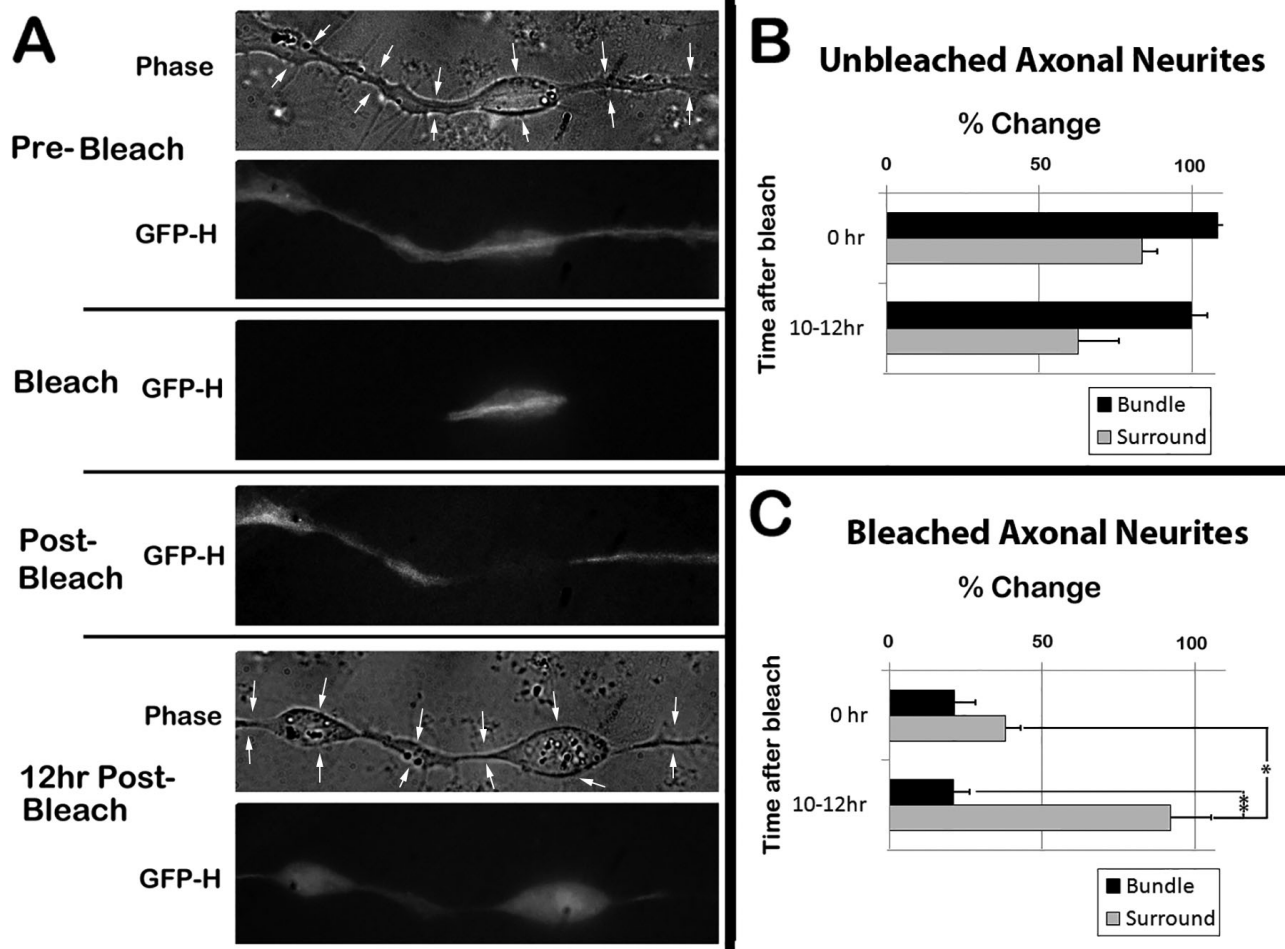
**Differential recovery of GFP within bundles and surrounding regions following photobleaching.** (A) Representative images of an Early-On cell 72 h after expression of GFP-H before, during, immediately after, and 12 h after photobleaching as described in the Materials and Methods. A region with a varicosity was selected to demonstrate more clearly recovery within the surround but not the bundle. Arrows in phase-contrast images denote the diameter of the axonal neurite. (B,C) Quantification of GFP in multiple unbleached and photobleached axonal neurites of the type presented in panel A. (*n*=5 cells for each condition; *P<0.05, **P<0.01).

Along these same lines, we reasoned that if we photobleached regions of neurites that had incorporated GFP-H in bundles during Early-On conditions, GFP would not readily re-incorporate into bundles (since these bundles were now established) but GFP may return to the surrounding regions. Early-On cells were photobleached 72 h after expression, and GFP-H was quantified immediately and 10-12 h after photobleaching (Fig. 7A). No statistical difference in GFP was detected over 10-12 h in unbleached axonal neurites or in regions of bleached neurites excluded from the bleached zone (Fig. 7A,B). Photobleaching significantly reduced GFP within the surround and bundles (Fig. 7A,C). By 10-12 h after photobleaching, GFP-H levels within the surround had returned to >90% of that present prior to photobleaching, yet GFP-H levels within the bundle remained statistically identical to the reduction immediately following photobleaching (Fig. 7A,C).

### Turnover of GFP-H

**Fig. 8.**
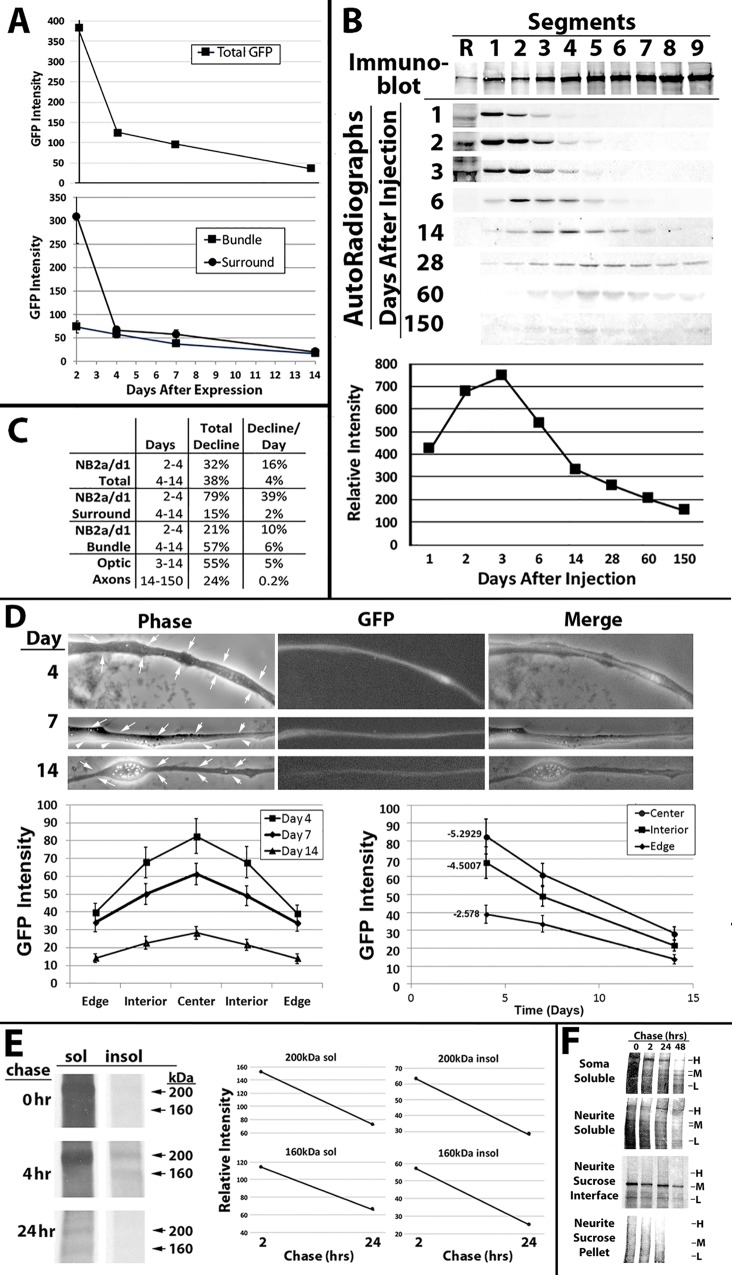
**Turnover of GFP-H and radiolabeled endogenous NF-H.** (A) Quantification of total GFP and GFP within the surround and bundle in Early-On cells. (B) Autoradiographs of NF-L within the retina (R) and 1.1 mm segments of optic axons at the indicated days following injection of ^35^S-methionine into the vitreous humor as described in the Materials and Methods. The accompanying graph presents quantification of radiolabeled NF-L from the above autoradiographs. (C) Quantification of the total decline and the rate of decline/day for GFP in NB2a/d1 cells and radiolabeled NFs in optic axons over the indicated intervals after expression or injection of radiolabel, respectively (*n*=72 cells, >10/time point, from three separate experiments). (D) Representative images of axonal neurites of Early-On cells at the indicated days after induction of differentiation. Arrows in phase-contrast images denote the diameter of the axonal neurite. The accompanying graphs present distribution of GFP across lateral profiles of bundles from central segment of multiple axonal neurites generated as described for Fig. 3 and in the Materials and Methods, and the rate of decline of GFP within the center, interior and edge, calculated as described in the Materials and Methods. (E) Autoradiographs of Triton-soluble (sol) and -insoluble (insol) fractions of differentiated NB2a/d1 cells at the indicated intervals after pulse-labeling with 35S-methionine as described in the Materials and Methods. The accompanying graphs present the relative intensity of nonphosphorylated (160 kDa) and extensively phosphorylated (200 kDa) at the indicated intervals after radiolabeling. (F) Autoradiographs of radiolabeled NFs from cells subjected to fractionation and sedimentation over sucrose as described in Fig. 4 at the indicated intervals (chase) following pulse-labeling with ^35^S-methionine as described in the Materials and Methods.

Expression of GFP-H for a limited window via an inducible promotor facilitated analysis of NF turnover. Overall GFP levels displayed a bi-phasic decline in Early-On cells. GFP declined rapidly for the first 2 days after expression, which was followed by a slower rate of decay from day 4-14 after expression. Quantification of levels in the bundle versus the surround revealed that this biphasic decline was confined to the surround. GFP levels within the bundle displayed a consistent slow rate of decline from day 2-14 after expression (Fig. 8A). The surround displayed the same slow rate of decline from days 4-14 after expression. This biphasic decline resembled that of radiolabeled NFs in optic pathway, where an initial rapid decline of the majority of newly-transported (radiolabeled) NFs within days was followed by a slower rate of decline over months (Fig. 8B,C) (Nixon and Logvinenko, 1986; Yuan et al., 2009; Jung et al., 2000a,b).

In efforts to monitor GFP-H turnover within bundles, we generated additional lateral profiles of bundles as carried out above to monitor incorporation of GFP (Fig. 3) at later times in culture (days 4, 7 and 14 in culture). At day 4, GFP intensity was highest within the center and intensity with the interior was higher than that on the edges and the rates of decline followed this same order (Fig. 8D). However, GFP displayed identical half-lives in each bundle region (6.5±0.1 days in the center, 6.2±0.3 in the interior, and 6.7±0.3 at the edge). Observation of identical half-lives suggests that the differential initial GFP intensity accounts for the apparent differential decay rates when plotted over days 4-14. In this regard, the center and interior display a biphasic decline. GFP itself has a half-life of ∼24 h, which can be modulated by conjugation to another protein sequence (Hazelrigg et al., 1998). Radiolabeling analyses indicated that NF-H itself underwent rapid turnover within these cells (Fig. 8E). These findings, coupled with the previous demonstration of similar rates of turnover of GPF-H (Szebenyi et al., 2002), indicates that incorporation of GFP-H into bundled NFs greatly extends its half-life.

Pulse-chase radiolabeling analyses have demonstrated incorporation of radiolabeled NFs throughout the axonal neurite cytoskeleton provided that ^35^S-methionine was added during axonal neurite outgrowth (Shea et al., 1990). Herein, we added radiolabel after 3 days of differentiation (by which time NF bundles have formed and outgrowth has slowed (Shea and Beermann, 1994; Yabe et al., 2000) and corresponding to Late-On GFP-H expression, then fractionated cultures as described in Fig. 4. Chase analyses demonstrated transport of radiolabeled NFs into axonal neurites and incorporation into the axonal neurite cytoskeleton, but, like GFP-H expressed under Late-On conditions, only trace amounts were recovered in the sucrose pellet, indicating that radiolabeled NFs were largely excluded from incorporation into the NF bundle (Fig. 8F).

## DISCUSSION

Considerable data from multiple laboratories indicate that NFs establish a stationary population that is maintained by continuous exchange with more rapidly transporting NFs/NF subunits (Nixon and Logvinenko, 1986; Rao et al., 2012; Yuan et al., 2015; Trivedi et al., 2007). Expression of GFP-tagged NF subunits in cultured neurons and neuronal cells demonstrated the establishment of a longitudinal bundle of closely-opposed, cross-linked NFs that were surrounded within axonal neurites of individual, more rapidly transporting NFs. Moreover, intact NF bundles can be biochemically separated from individual NFs in preparations from spinal cord and sciatic nerve as well as neuronal cultures (Yabe et al., 2001a,b; Kushkuley et al., 2009). Bundled NFs are thought to correspond to the stationary NFs observed following radiolabeling *in situ* (Yabe et al., 2001a,b; Yuan et al., 2009, 2012; Shea et al., 2009; Lee and Shea, 2012). NF bundles form when a sufficient concentration of NFs is present, and furthermore requires NF-H, which explains why bundled NFs are not observed not all culture systems or axons *in situ* since some systems lack appreciable concentrations of NFs, and/or NF-H (Yuan et al., 2009; Boumil et al., 2015; Lee and Shea, 2014).

The relatively rapid saturation of the axonal neurite cytoskeleton with GFP-tagged subunits hindered more detailed analyses of NF dynamics in prior studies with continuous expression of GFP-H. Herein, expression of GFP-H under the control of an inducible promoter facilitated these analyses, since we were able to express GFP-H for a short window of time at different periods (i.e. ‘Early-On’ and ‘Late-On’) during axonal neurite outgrowth. GFP-H rapidly translocated into and along axonal neurites at identical levels under both conditions of expression. However, Early-On cells incorporated significantly more GFP into NF bundles than did Late-On cells. NF bundles are prominent within the axonal neurites of NB2a/d1 cells by 72 h after induction of differentiation (Yabe et al., 2001a,b). Since Early-On cells expressed and transported GFP-H into axons from 24-36 h after induction of differentiation, a substantial pool of GFP-H was present during formation of, and consequently was incorporated into, NF bundles. By contrast, Late-On cells did not express and transport GFP-H until 72-84 h after induction of differentiation, which is after NF bundles had already formed (Yabe et al., 2001a,b). Consequently, NF bundles in Late-On cells would be formed from endogenous, untagged NF-H. These conclusions were supported by additional manipulation and observation as follows: (1) regions of NF bundles of Early-On cells that were photobleached following cessation of GFP-H expression did not recover fluorescence while the surround did (likely since photobleached neurites now essentially corresponded to bundles of Late-On cells), and (2) bundles within the most distal regions of axonal neurites were prominently labeled with GFP (likely since, unlike proximal and central regions, distal regions were still growing during Late-On expression and therefore corresponded to bundles in Early-On cells).

GFP levels declined rapidly within the area of axonal neurites surrounding NF bundles in both Early-On and Late-On cells, but displayed a much slower decline within bundles. This differential decline between individual and bundled NFs, coupled with differential incorporation of NFs into bundles following Early-On and Late-On expression suggested that bundled NFs are more stable than NF not incorporated into bundles.

GFP-H levels within bundles of Early-On cells declined substantially and continuously during continued incubation. By contrast, the relatively lower levels of GFP-H in bundles of Late-On cells remained more constant over this interval. The levels of GFP associated with bundles and with the surround of cells under both conditions converged by 2 weeks in culture. The eventual convergence of the rate of GFP decline of GFP in the surround and bundle after expression was halted is consistent with continued exchange between bundles and surrounding NFs (Yabe et al., 2001a,b). This biphasic decay resembles that observed in optic pathway (Nixon and Logvinenko, 1986; Jung et al., 2000a,b). Our findings suggest that the surround, which includes Triton-soluble as well as -insoluble NFs, may represent the rapidly-decaying NFs observed in optic axons, while bundled correspond to the slow-decaying population (Nixon and Logvinenko, 1986; Jung and Shea, 1999; Millecamps et al., 2007; Yuan et al., 2009).

It has been suggested that the biphasic decline of radiolabeled NF subunits along axons *in situ* did not reveal the establishment of a stationary phase (Nixon and Logvinenko, 1986), but rather that the initial rapid decay was derived from co-migrating contaminants (Brown and Jung, 2013). Notably, our observation of the identical biphasic dynamics for radiolabeled NF subunits immunoprecipitated from optic pathway by an anti-NF antibody eliminates this alternative possibility.

Bundles isolated from cultures and axons *in situ* remain intact and retain NF phospho-epitopes for >7 days but dissociated into individual NFs following addition of EDTA/EGTA or phosphatases. Notably, this dissociation required 4-7 days (Kushkuley et al., 2009). These features additional evidence that NF bundles are relatively stable structures. Microtubules are excluded from NF bundles both within intact cell and axons *in situ*, and this exclusion persists following isolation of bundles from cultures and axons *in situ* (Kushkuley et al., 2009). Since microtubule motors translocate NFs, it was suggested NFs could essentially become ‘trapped’ within the interior of the bundle, giving rise to a population of extremely long-lived NFs, while exchange could continue only at the edges of bundles (Shea and Yabe, 2000). The findings of the present study are consistent with this line of reasoning.

Consideration of the NF bundle as a relatively stable NF population was further supported by consideration of half-lives of GFP and of NF subunits in these cells. Non-conjugated GFP has a half-life of ∼24 h (Hazelrigg et al., 1998). Triton-soluble and -insoluble NF-H, including that which had transported into axonal neurites but was not incorporated into bundles, also displayed a half-life of ≤24 h as shown herein. Prior studies demonstrated that GFP-H decayed by ∼50% within 2 days following cessation of expression; notably, however, GFP-H mRNA persisted relatively unchanged for 12 h before declining, suggesting that the half-life of GFP-H itself was closer to 1 day (Szebenyi et al., 2002). By contrast, GFP-H within bundles displayed a half-life of ∼6 days, indicating that bundled NFs persisted at least six times longer than non-bundled NFs. The identical half-life of signal within the center and edges of bundles suggests that decay of fluorescence, rather than NF/NF subunit exchange was responsible for the ultimate depletion of GFP signal from the ‘core’ of bundles in Early-On cells. Accordingly, the 6-day half-life may underestimate the stability of bundled NFs.

The stationary pool of NFs is generated by progressive phospho-dependent NF-NF associations. While extensive phosphorylation may initially be considered to foster repulsion between NFs, extensively phosphorylated C-terminal extensions instead form cation-dependent cross-bridges (Kushkuley et al., 2009). NF C-terminal phosphorylation does not directly impede axonal transport, but does so indirectly by fostering NF-NF associations that generate a macro-structure too large to undergo transport ‘resident’ population that undergoes continuous exchange with more rapidly transporting NFs (Shea and Lee, 2011). In this regard, the stationary phase *in situ* and NF bundles in culture are comprised of the most highly-phosphorylated NFs (Lewis and Nixon, 1988; Yabe et al., 2001a,b). Phosphorylation of the NF-H C-terminal domain protects NFs from proteolysis (Goldstein et al., 1987; Pant, 1988; Greenwood et al., 1993) and promotes divalent cation-mediated associations between NFs and with other cytoskeletal elements. In doing so, phosphorylation increased overall levels of axonal NFs and maintained the stationary phase *in situ* and promoted NF bundling within axonal neurites (Rao et al., 2012; Lee et al., 2014).

Following isolation, bundled and individual NFs could be interconverted by modulation of phosphorylation. Bundled NFs were readily dissociated by calcium chelation or phosphatase treatment, which depleted phospho-dependent, divalent cation-mediated crosslinking, while incubation with known NF kinases induced NF-NF associations among individual NFs. Moreover, NFs released following dissolution of isolated bundles underwent kinesin-mediated association with MTs and displayed MT-dependent motility in cell-free assays to the same degree as those NFs recovered as individual NFs, while bundles did not translocate in motility assays (Kushkuley et al., 2009; Lee et al., 2011b). These findings provide additional evidence that bundled NFs represent stationary macro-structures, and furthermore support that there is phospho-dependent exchange between these two pools of NFs. The dynamics of NF transport have been mathematically modeled (Brown et al., 2005; Craciun et al., 2005). This model describes a pool of NFs associated with motors (‘on’), a pool that is dissociated from motors (‘off’) but can readily re-associate with them, and a third pool (‘away’) that is in some manner restricted from entering the pool of NFs which can reversibly associate with motors (Brown et al., 2005; Craciun et al., 2005; Trivedi et al., 2007; Kuznetsov and Kuznetsov, 2013). The findings of the present study provide further evidence that phospho-mediated formation of NF-NF associations, which withdraw NFs at least transiently from the transporting pool (Lee et al., 2011a; Kushkuley et al., 2009; Sunil et al., 2012; Yabe et al., 2001a,b), represents the pool of NFs that is characterized as ‘away’ in modeling.

Intermediate filaments in general provide structural support and tensile strength to cells (Fuchs, 1994; Helfand et al., 2004). Perhaps nowhere can such support be more critical than along axons, which can be thousands of times longer than their cell bodies and which lack desomosomes or other interconnections that mediate regional support among other cell types. Unlike other cells, however, neurons are faced with the challenge of orderly transport and assembly of NFs within axons, and errors in this process can be catastrophic (Rao and Nixon, 2003; Yuan et al., 2017). The complexity of this process, which encompasses multiple motor proteins, kinases, phosphatases and proteases, can preclude consideration of the function of NFs beyond transport itself (Lee et al., 2014; Shea and Flanagan, 2001; Shea and Yabe, 2000). Development and maintenance of a functional nervous system is by definition dependent upon orderly elaboration and maintenance of the axons, which in turn is dependent upon organization of the axonal cytoskeleton. NFs provide the stabilizing force during axonal pathfinding and outgrowth as well as for axonal maintenance following synaptogenesis (Yuan et al., 2017). The establishment of a stationary population minimizes NF turnover, which would otherwise impart a prohibitive metabolic burden upon the neuron (Nixon and Logvinenko, 1986). Moreover, a stationary NF network is likely important to mediate anchoring of receptor proteins and organelles within axons (Ehlers et al., 1998; Kim et al., 2002; Ratnam and Teichberg, 2005). The findings herein and in prior studies (Yabe et al., 2009; Kushkuley et al., 2009; Yuan et al., 2009) indicate that bundled NFs represent this stationary network in cultured neurons and neuronal cells. In this regard, such structures, and their formation by multivalent cations, are by no means unique to NFs. Bundles are formed by multiple intermediate filament proteins, including vimentin, keratin, desmin and Glial Fibrillary Acidic Protein (Perng et al., 1999; Franke et al., 1978; Tokuyasu et al., 1983; Wagner et al., 2007), and an increase in bundles accompanies a requirement for extra stabilization (Bornslaeger et al., 1996; Djabali et al., 1999; Jones et al., 1982). Further studies of NF dynamics may be facilitated by the use of an inducible promoter such as that utilized herein.

## MATERIALS AND METHODS

### Establishment of a tetracycline-inducible system for differential expression of NF-H

An N-terminally GFP-tagged *Rattus norvegicus* neurofilament heavy (GFP-H) cDNA sequence housed in a pEGFP-C3 vector (Boumil et al., 2015; Lee et al., 2014; Ackerley et al., 2003) was digested by BamHI (New England Biolabs, Ipswich, MA, USA) at a single restriction site and converted to blunt ends by Klenow (New England Biolabs) and purified by a PCR cleanup kit (Qiagen, Germantown, MD, USA). Extracted linear DNA was then digested by AgeI (New England Biolabs). The resulting fragments were separated by 0.8% agarose gel electrophoresis and the fragment containing the GFP-H cDNA was excised and purified by a gel extraction kit (Qiagen). A pcDNA5 vector (Thermo Fisher Scientific) was prepared by the same enzyme and purification treatments. The GFP-H cDNA was then ligated into the pcDNA5 vector by means of T4 DNA ligase (New England Biolabs) at a 3:1 insert:vector ratio. 5α-competent *E. coli* (New England Biolabs) was transformed with the resulting pcDNA5-GFP-H.

### Cell culture and transfection

Mouse NB2a/d1 neuroblastoma cells were maintained in high-glucose DMEM supplemented with 10% tet-tested fetal bovine serum (Atlanta Biologicals, Flowery Branch, GA, USA), 2 mM L-glutamine, and antibiotic-antimycotic solution (‘complete DMEM’) and differentiated for 20 h with 1 mM dibutyrl-cyclic-AMP (Yabe et al., 1999). It is recognized that these differentiated neuroblastoma may lack key features of bona fide neurons, so all conclusions must be viewed with caution. However, studies spanning decades have shown these cells to be an ideal model for analyses of NF dynamics since they robustly express all NF subunits, phosphorylate them via known NF kinases, transport them into and along axonal neurites via the same motors as *in situ*; finally, they are readily transfected and large quantities are easily obtained for comparative biochemical and radiolabeling analyses (Boumil et al., 2015; Chan et al., 2003, 2004, 2005; Dubey et al., 2007; Kushkuley et al., 2009; Lee and Shea, 2014; Lee et al., 2011a,b, 2014; Moran et al., 2005; Motil et al., 2006, 2007; Sunil et al., 2012; Vohnoutka et al., 2017; Yabe et al., 1999, 2001a,b). Cultures were transiently transfected pcDNA5-GFP-H and its accompanying pcDNA6 construct via PolyJet (Signagen, Rockville, MD, USA) for 4 h in the presence of 0.5 µg/ml doxycycline (Qin et al., 2010) as follows: (1) doxycycline for 12 h immediately after transfection (‘Early-On’); (2) doxycycline for 12 h commencing 48 h after transfection (‘Late-On’); (3) doxycycline for 72 h (‘Always-On’); (4) no doxycycline for the duration of the assay (‘Leak’).

Expression was allowed to continue for 12 h since this is the minimum time to obtain as seen with this construct (Yabe et al., 2001a,b).

### Immunofluorescence and photobleaching

Cells cultivated and transfected on acid-washed poly-d-lysine/laminin coated glass coverslips housed within petri dishes or glued below a hole drilled in the bottom of the dish (Whitlon and Baas, 1992) were incubated for 2 h at room temperature in PBS containing 2% goat serum and 0.2% Triton and either a 1:1000 dilution of monoclonal antibody SMI31 (diluted 1:1000) or a 1:100 dilution of monoclonal antibody RT97, both of which are directed against NF-H C-terminal phosphoepitopes (Yabe et al., 2001a,b). Following primary antibody incubation and washing, cultures were rinsed in the same buffer without antibodies then incubated for 1 h at room temperature in the same buffer containing a 1:500 dilution of rhodamine-conjugated goat anti-mouse IgG, dehydrated in a series of increasing ethanol concentrations (70/80/95/100%), rinsed twice in xylene, and mounted on glass slides with DePeX. Fluorescence intensity and localization was monitored using a Zeiss Axiovert microscope under FITC or TRITC optics (for Rhodamine-conjugated secondary antibodies) along with corresponding phase contrast images. Sequential mages through the vertical plane of axonal neurites with respect to the culture dish were captured at 100 nm intervals via Velocity 5.4.1 software, and the resulting Z-stacks were deconvoluted with the same software (Moran et al., 2005).

Regions of axonal neurites were photobleached by closing the aperture diaphragm and exposing select portions of axonal neurites to the excitation beam for 2 min, which dramatically depletes GFP fluorescence (Chan et al., 2005; Motil et al., 2006; Wang and Brown, 2001). Cells were imaged prior to, immediately following, and 10-12 h after photobleaching.

### Image analysis

Image analysis was carried out using ImageJ (https://imagej.nih.gov/ij/:), and recorded values were imported into Microsoft Excel. GFP was quantified within total axonal neurites and within proximal, central, and distal axonal segments of equivalent length, in five separate experiments. GFP was also quantified within centrally-situated, longitudinally-oriented NF bundles, and within the surrounding neurite cytoplasm by outlining a region of the bundle with the free-hand selection tool, then moving the same selection box to the adjacent cytoplasm as monitored in fluorescent as well as corresponding phase-contrast images (Yabe et al., 2001a,b; Chan et al., 2005). Phase-contrast images were included to allow assessment of neurite morphology independent of any fluorescent signal and therefore facilitate differential distribution of GFP within neurites.

The half-life of GFP was calculated by determining the decay coefficient (λ) in the exponential decay equation N_(T)_=N_o_ e^λt^, where N_o_ is the initial quantity of signal detected, *N*_(T)_ represents the quantity of signal detected after a given time has elapsed (t), e represents the natural exponential, and λ representing the decay coefficient. The half-life of GFP was calculated by T_(1/2)_=ln(2)/λ, where T_(1/2)_ represents half-life, and λ represents the decay coefficient.

Immunofluorescent intensity was analyzed by generating lateral fluorescence intensity profiles across the neurites using the Plot Profile feature. Values for the neuritic cytoplasm on both sides of the centrally-situated NF bundle (‘surround’) were averaged and subtracted from the respective value of the NF bundle. In order to normalize values among bundles from different axonal neurites to monitor GFP incorporation, regions within the NF bundle were then grouped into five equivalently-sized transverse bins (‘edge’, ‘interior’, ‘center’, ‘interior’ and ‘edge’) and fluorescent intensity was averaged within each of these bins to generate five data points for each NF bundle. Values corresponding to the edges were averaged and the five bins were divided by this average to generate a distribution profile for each NF bundle. To monitor GFP decay within bundles, values within these five transverse binds were not divided by the average of the edges, to allow comparison of decay rates among all five bins. Statistical comparisons were carried out using Student's unpaired, two-tailed, *t*-test. Notably, while GFP was only expressed conjugated to NF-H and not in isolation, we refer exclusively to GFP, rather than GFP-H in immunofluorescent analyses, since we cannot be certain that some of the GFP signal is derived from free GFP or GFP conjugated to NF-H fragments due to proteolysis (although Triton-extraction and biochemical analyses indicate that any such population within axonal neurites is small).

### Fractionation of cells

Cultures were scraped from plates and lysed in 50 mM Tris containing 2 mM EDTA, Complete™ protease inhibitor cocktail (Roche), PhosSTOP™ phosphatase inhibitor cocktail (Roche) and 1 mM PMSF. Lysates were gently homogenized with a loose-fitting Teflon pestle, and centrifuged at 500× ***g*** for 5 min, which sediments axonal neurite fragments and disrupts cell somae (Shea et al., 1993). The resulting supernatant (‘soma’ fraction) was decanted and brought to a final Triton X-100 concentration of 1%, and the pellet (‘axonal neurite’ fraction) was resuspended in the above buffer with 1% Triton. The resuspended axonal neurite fraction was homogenized with a tight-fitting Teflon pestle, and centrifuged at 13,100× ***g*** for 15 min over 1 ml of a 1 M sucrose cushion. Prior studies confirmed that NF bundles sediment through the sucrose cushion (‘sucrose pellet’), individual and loosely-associated NFs are retained on the top of the sucrose cushion (‘interface’) and that the supernatant above the interface (‘soluble’ fraction) contains unassembled NF subunis and small oligomeric NF assemblies; Yabe et al., 2001a,b; Shea et al., 1993). These interface and sucrose pellet fractions were resuspended in the above buffer containing 8 M urea to dissociate bundled NFs; Leterrier and Eyer, 1987; Kushkuley et al., 2009). The soma fraction was centrifuged at 13,100× ***g*** for 15 min. The resultant supernatant (‘soma soluble’) was decanted and the pellet resuspended in the above buffer containing 8 M urea (‘soma insoluble’). Some lysates were analyzed by phase-contrast and fluorescence microscopy prior to and following homogenization, and after 500× ***g*** centrifugation, to confirm partitioning of axonal neurite fragments (Shea et al., 1993).

Fractions were subjected to sodium dodecyl sulfate polyacrylamide gel electrophoresis (SDS-PAGE) using 4-15% acrylamide gradient gels and transferred to nitrocellulose membranes in 25 mM Tris-HCl (pH 7.6) containing 192 mM glycine and 10% methanol. Membranes were blocked in 50 mM Tris-HCl (pH 7.6) containing 154 mM NaCl, 0.1% Tween-20 (TBST) containing 5% goat serum and 1% bovine serum albumin. Membranes were incubated in the following primary antibodies in TBST with 2% goat serum overnight at 4°C: a 1:500 dilution of an antibody (R39) that reacts with all NF subunits regardless of phosphorylation state (Jung and Shea, 1999), a 1:2000 dilution of a monoclonal antibody (SMI32) that reacts with a phospho-epitope of NF-H and NF-M when that epitope is not phosphorylated (Covance, Princeton, NJ, USA), a 1:1000 dilution of SMI31 (Covance), a 1:100 dilution of RT97, a 1:2000 dilution of an antibody (DM1A) that reacts with α-tubulin (Santa Cruz Biotechnology), a 1:1000 dilution of an antibody (6-11B-1) that reacts with acetylated α-tubulin (Abcam), a 1:1000 dilution of an antibody (Tau46) that reacts with tau, a 1:10,000 dilution of an antibody that reacts with MAP2 (Thermo-Fisher Scientific), and a 1:1000 dilution of an antibody that reacts with the soluble glycolytic enzyme glyceraldehyde-3-phosphate dehydrogenase (GAPDH; Cell Signaling Technology). Membranes were washed three times for 5 min/wash in TBST, and incubated in the appropriate goat alkaline phosphatase-conjugated secondary IgG for 1 h at room temperature. Membranes were washed three times for 5 min/wash in TBST, and once for 5 min in 50 mM Tris (pH 7.6) containing 154 mM NaCl. Membranes were washed a final time in 100 mM Tris (pH 9.5) containing 100 mM NaCl and 5 mM MgCl_2_ for 7 min, and immune-labeled proteins visualized by incubation in the same buffer also containing 1% 5-bromo-4-chloro-3′-indolyphosphate and 1.5% nitro-blue tetrazolium. Scanned images of membranes were analyzed by ImageJ software. Statistical analyses were carried out using Student's *t*-test.

### Radiolabeling of NFs *in situ* and monitoring of transport and exchange

Murine retinal ganglion cells of normal mice were radiolabeled *in situ* by injection of 70 µCi ^35^S-methionine in a total volume of 0.2 µl via a pulled glass capillary pipette into the vitreous of anesthetized mice (Nixon and Logvinenko, 1986). Mice were sacrificed by cervical dislocation at 1-150 days following injection. Retinas were dissected away from the rest of the eye and optic axons dissected into 9×1.1 mm segments on a glass slide on dry ice. Retinas and segments from 5-11 mice were pooled and homogenized in 1% Triton X-100 in 50 mM Tris (pH 6.9) containing 2 mM EDTA, 1 mM PMSF and 50 µg/ml leupeptin at 4°C by 50 strokes in a tight-fitting glass-Teflon homogenizer (Jung and Shea, 1999; Nixon and Logvinenko, 1986). The Triton-insoluble cytoskeleton was sedimented by centrifugation 15,000× ***g*** for 15 min as described (Shea et al., 1990). NF subunits were immunoprecipitated from the above fractions with a 1:150 dilutions of a polyclonal antibody that quantitatively immunoprecipitates all three NF subunits (R39) Immunoprecipitated material was subjected to SDS-gel electrophoresis, dried and transferred to nitrocellulose. Some replicas were probed with R39 and alkaline phosphatase-conjugated secondary antibody as above, and others were placed against Kodak X-Omat film to generate autoradiographs. Radiolabeled data presented for normal mice from day 1-60 appeared previously in Jung and Shea (1999) and are reproduced (with permission) along with novel data from day 150 after injection to facilitate comparison of subunit transport and exchange/decay over this protracted interval. Autoradiographs were digitized via a UMax scanner equipped with a transparency adaptor operated by a Macintosh. Densitometric analyses of digitized images were carried out via ImageJ software by encircling the entire band with the program's freehand selection tool. Since the NF triplet co-migrated along optic axons, we subsequently present densitometric data only for NF-L for simplicity only (Jung and Shea, 1999). Densitometric calculations using the NF triplet yielded identical relative distributions (data not shown; Yabe et al., 2000). Total mean density (density per total area of immunoreactive band) was calculated for autoradiographs. As shown previously (Jung and Shea, 1999), all radiolabel associated with NFs within cytoskeletons had transported into axons after day 3. To facilitate comparison of distribution and loss of radiolabeled subunits at different post-injection intervals, total radiolabel associated with NF-L within axons at day 3 was defined as 100% and the amount remaining within axons at subsequent days was compared to this level.

## References

[BIO028795C1] Ackerley S., Thornhill P., Grierson A. J., Brownlees J., Anderton B. H., Leigh P. N., Shaw C. E. and Miller C. C. J. (2003). Neurofilament heavy chain side arm phosphorylation regulates axonal transport of neurofilaments. *J. Cell Biol.* 161, 489-495. 10.1083/jcb.200303138 12743103 PMC2172950

[BIO028795C2] Alami N. H., Jung P. and Brown A. (2009). Myosin Va increases the efficiency of neurofilament transport by decreasing the duration of long-term pauses. *J. Neurosci.* 29, 6625-6634. 10.1523/JNEUROSCI.3829-08.2009 19458233 PMC2943491

[BIO028795C3] Bornslaeger E. A., Corcoran C. M., Stappenbeck T. S. and Green K. J. (1996). Breaking the connection: displacement of the desmosomal plaque protein desmoplakin from cell-cell interfaces disrupts anchorage of intermediate filament bundles and alters intercellular junction assembly. *J. Cell Biol.* 134, 985-1001. 10.1083/jcb.134.4.985 8769422 PMC2120955

[BIO028795C4] Boumil E., Vohnoutka R., Lee S. and Shea T. B. (2015). Early expression of the high molecular weight neurofilament subunit attenuates axonal neurite outgrowth. *Neurosci. Lett.* 604, 36-41. 10.1016/j.neulet.2015.07.031 26225928

[BIO028795C5] Brown A. and Jung P. (2013). A critical reevaluation of the stationary axonal cytoskeleton hypothesis. *Cytoskeleton* 70, 1-11. 10.1002/cm.21083 23027591 PMC3725768

[BIO028795C6] Brown A., Wang L. and Jung P. (2005). Stochastic simulation of neurofilament transport in axons: the “stop-and-go” hypothesis. *Mol. Biol. Cell* 16, 4243-4255. 10.1091/mbc.E05-02-0141 16000374 PMC1196334

[BIO028795C7] Chan W. K.-H., Yabe J. T., Pimenta A. F. and Shea T. B. (2003). Growth cones contain a dynamic population of neurofilament subunits. *Cell Motil. Cytoskel.* 54, 195-207. 10.1002/cm.10084 12589678

[BIO028795C8] Chan W. K.-H., Dickerson A., Otriz D., Pimenta A. F., Moran C. M., Motil J., Snyder S. J., Pant H. C. and Shea T. B. (2004). Mitogen-activated protein kinase regulates neurofilament axonal transport. *J. Cell Sci.* 117, 4629-4642. 10.1242/jcs.01135 15331628

[BIO028795C9] Chan W. K.-H., Yabe J. T., Pimenta A. F., Ortiz D. and Shea T. B. (2005). Neurofilaments can undergo axonal transport and cytoskeletal incorporation in a discontinuous manner. *Cell Motil. Cytoskeleton* 62, 166-179. 10.1002/cm.20089 16211584

[BIO028795C10] Craciun G., Brown A. and Friedman A. (2005). A dynamical system model of neurofilament transport in axons. *J. Theor. Biol.* 237, 316-322. 10.1016/j.jtbi.2005.04.018 15975597 PMC1995014

[BIO028795C11] Djabali K., Piron G., de Néchaud B. and Portier M.-M. (1999). αB-crystallin interacts with cytoplasmic intermediate filament bundles during mitosis. *Exp. Cell Res.* 253, 649-662. 10.1006/excr.1999.4679 10585288

[BIO028795C12] Dubey M., Chaudhury P., Kabiru H. and Shea T. B. (2007). Tau inhibits anterograde axonal transport and perturbs stability in growing axonal neurites in part by displacing kinesin cargo: neurofilaments attenuate tau-mediated neurite instability. *Cell Motil. Cytoskeleton* 65, 89-99. 10.1002/cm.20243 18000878

[BIO028795C13] Ehlers M. D., Fung E. T., O'Brien R. J. and Huganir R. L. (1998). Splice variant-specific interaction of the NMDA receptor subunit NR1 with neuronal intermediate filaments. *J. Neurosci.* 18, 720-730. 9425014 10.1523/JNEUROSCI.18-02-00720.1998 PMC6792537

[BIO028795C14] Franke W. W., Schmid E., Osborn M. and Weber K. (1978). Different intermediate-sized filaments distinguished by immunofluorescence microscopy. *Proc. Natl. Acad. Sci. USA* 75, 5034-5038. 10.1073/pnas.75.10.5034 368806 PMC336257

[BIO028795C15] Fuchs E. (1994). Intermediate filaments and disease: mutations that cripple cell strength. *J. Cell Biol.* 125, 511-516. 10.1083/jcb.125.3.511 7513705 PMC2119985

[BIO028795C16] Goldstein M. E., Sternberger N. H. and Sternberger L. A. (1987). Phosphorylation protects neurofilaments against proteolysis. *J. Neuroimmunol.* 14, 149-160. 10.1016/0165-5728(87)90049-X 3029175

[BIO028795C17] Gotow T. and Tanaka J. (1994). Phosphorylation of neurofilament H subunit as related to arrangement of neurofilaments. *J. Neurosci. Res.* 37, 691-713. 10.1002/jnr.490370604 8046771

[BIO028795C18] Gotow T., Tanaka T., Nakamura Y. and Takeda M. (1994). Dephosphorylation of the largest neurofilament subunit protein influences the structure of crossbridges in reassembled neurofilaments. *J. Cell Sci.* 107, 1949-1957. 7983161 10.1242/jcs.107.7.1949

[BIO028795C19] Greenwood J. A., Troncoso J. C., Costello A. C. and Johnson G. V. W. (1993). Phosphorylation modulates calpain-mediated proteolysis and calmodulin binding of the 200-kDa and 160-kDa neurofilament proteins. *J. Neurochem.* 61, 191-199. 10.1111/j.1471-4159.1993.tb03555.x 8515266

[BIO028795C20] Hazelrigg T., Liu N., Hong Y. and Wang S. (1998). GFP expression in *Drosophila* tissues: time requirements for formation of a fluorescent product. *Dev. Biol.* 199, 245-249. 10.1006/dbio.1998.8922 9698444

[BIO028795C21] Helfand B. T., Chang L. and Goldman R. D. (2004). Intermediate filaments are dynamic and motile elements of cellular architecture*.* *J. Cell Sci.* 117:133-141. 10.1242/jcs.00936 14676269

[BIO028795C22] Hirokawa N., Glicksman M. A. and Willard M. B. (1984). Organization of mammalian neurofilament polypeptides within the neuronal cytoskeleton. *J. Cell Biol.* 98, 1523-1536. 10.1083/jcb.98.4.1523 6425303 PMC2113240

[BIO028795C23] Hisanaga S.-I. and Hirokawa N. (1988). Structure of the peripheral domains of neurofilaments revealed by low angle rotary shadowing. *J. Mol. Biol.* 202, 297-305. 10.1016/0022-2836(88)90459-7 3172218

[BIO028795C25] Jones J. C. R., Goldman A. E., Steinert P. M., Yuspa S. and Goldman R. D. (1982). Dynamic aspects of the supramolecular organization of intermediate filament networks in cultured epidermal cells. *Cytoskeleton* 2, 197-213. 10.1002/cm.970020302 6756644

[BIO028795C26] Jung C. and Shea T. B. (1999). Regulation of neurofilament axonal transport by phosphorylation in optic axons in situ. *Cell Motil. Cytoskeleton* 42, 230-240. 10.1002/(SICI)1097-0169(1999)42:3<230::AID-CM6>3.0.CO;2-A 10098936

[BIO028795C28] Jung C., Yabe J. T., Lee S. and Shea T. B. (2000a). Hypophosphorylated neurofilament subunits undergo axonal transport more rapidly than more extensively phosphorylated subunits in situ. *Cell Motil. Cytoskeleton* 47, 120-129. 10.1002/1097-0169(200010)47:2<120::AID-CM3>3.0.CO;2-6 11013392

[BIO028795C29] Jung C., Yabe J. T. and Shea T. B. (2000b). C-terminal phosphorylation of the high molecular weight neurofilament subunit correlates with decreased neurofilament axonal transport velocity. *Brain Res.* 856, 12-19. 10.1016/S0006-8993(99)02314-8 10677606

[BIO028795C30] Jung C., Chylinski T. M., Pimenta A., Ortiz D. and Shea T. B. (2004). Neurofilament transport is dependent on actin and myosin. *J. Neurosci.* 24, 9486-9496. 10.1523/JNEUROSCI.1665-04.2004 15509735 PMC6730143

[BIO028795C31] Jung C., Lee S., Ortiz D., Zhu Q., Julien J.-P. and Shea T. B. (2006). The high and middle molecular weight neurofilament subunits regulate the association of neurofilaments with kinesin: Inhibition by phosphorylation of the high molecular weight subunit. *Mol. Brain Res.* 141, 151-155. 10.1016/j.molbrainres.2005.08.009 16246456

[BIO028795C32] Kim O. J., Ariano M. A., Lazzarini R. A., Levine M. S. and Sibley D. R. (2002). Neurofilament-M interacts with the D1 dopamine receptor to regulate cell surface expression and desensitization. *J. Neurosci.* 22, 5920-5930. 12122054 10.1523/JNEUROSCI.22-14-05920.2002 PMC6757921

[BIO028795C33] Kushkuley J., Chan W. K. H., Lee S., Eyer J., Leterrier J.-F., Letournel F. and Shea T. B. (2009). Neurofilament cross-bridging competes with kinesin-dependent association of neurofilaments with microtubules. *J. Cell Sci.* 122, 3579-3586. 10.1242/jcs.051318 19737816

[BIO028795C34] Kuznetsov I. A. and Kuznetsov A. V. (2013). Analytical comparison between Nixon-Logvinenko's and Jung-Brown's theories of slow neurofilament transport in axons. *Math. Biosci.* 245, 331-339. 10.1016/j.mbs.2013.08.001 23958382

[BIO028795C35] Lee S. and Shea T. B. (2012). The discontinuous nature of neurofilament transport accommodates both establishment and repair of the axonal neurofilament array. *Cytoskeleton* 70, 67-73. 23124969 10.1002/cm.21087

[BIO028795C36] Lee S. and Shea T. B. (2014). The high molecular weight neurofilament subunit plays an essential role in axonal outgrowth and stabilization. *Biol. Open* 3, 974-981. 10.1242/bio.20149779 25260918 PMC4197446

[BIO028795C37] Lee S., Sunil N. and Shea T. B. (2011a). C-terminal neurofilament phosphorylation fosters neurofilament-neurofilament associations that compete with axonal transport. *Cytoskeleton* 68, 8-17. 10.1002/cm.20488 20862740

[BIO028795C38] Lee S., Sunil N., Tejada J. M. and Shea T. B. (2011b). Differential roles of kinesin and dynein in translocation of neurofilaments into axonal neurites. *J. Cell Sci* 124, 1022-1031. 10.1242/jcs.079046 21363889

[BIO028795C39] Lee S., Pant H. C. and Shea T. B. (2014). Divergent and convergent roles for kinases and phosphatases in neurofilament dynamics. *J. Cell Sci.* 127, 4064-4077. 10.1242/jcs.153346 25015294 PMC6519427

[BIO028795C41] Leterrier J.-F. and Eyer J. (1987). Properties of highly viscous gels formed by neurofilaments in vitro. A possible consequence of a specific inter-filament cross-bridging. *Biochem J.* 245, 93-101. 10.1042/bj2450093 3663160 PMC1148086

[BIO028795C42] Lewis S. E. and Nixon R. A. (1988). Multiple phosphorylated variants of the high molecular mass subunit of neurofilaments in axons of retinal cell neurons: characterization and evidence for their differential association with stationary and moving neurofilaments. *J. Cell Biol.* 107, 2689-2701. 10.1083/jcb.107.6.2689 3144556 PMC2115653

[BIO028795C43] Liem R. K., Yen S. H., Salomon G. D. and Shelanski M. L. (1978). Intermediate filaments in nervous tissues. *J. Cell Biol.* 79, 637-645. 10.1083/jcb.79.3.637 83322 PMC2110269

[BIO028795C44] Millecamps S., Gowing G., Corti O., Mallet J. and Julien J.-P. (2007). Conditional NF-L transgene expression in mice for in vivo analysis of turnover and transport rate of neurofilaments. *J. Neurosci.* 27, 4947-4956. 10.1523/JNEUROSCI.5299-06.2007 17475803 PMC6672085

[BIO028795C45] Moran C. M., Donnelly M., Ortiz D., Pant H. C., Mandelkow E.-M. P. and Shea T. B. (2005). Cdk5 inhibits anterograde axonal transport of neurofilaments but not that of tau by inhibition of mitogen-activated protein kinase activity. *Mol. Brain Res.* 134, 338-344. 10.1016/j.molbrainres.2004.10.035 15836929

[BIO028795C46] Motil J., Chan W. K.-H., Dubey M., Chaudhury P., Pimenta A., Chylinski T. M., Ortiz D. T. and Shea T. B. (2006). Dynein mediates retrograde neurofilament transport within axons and anterograde delivery of NFs from perikarya into axons: regulation by multiple phosphorylation events. *Cell Motil. Cytoskeleton* 63, 266-286. 10.1002/cm.20122 16570247

[BIO028795C47] Motil J., Dubey M., Chan W. K.-H. and Shea T. B. (2007). Inhibition of dynein but not kinesin induces aberrant focal accumulation of neurofilaments within axonal neurites. *Brain Res.* 1164, 125-131. 10.1016/j.brainres.2006.09.108 17640622

[BIO028795C48] Nixon R. A. and Logvinenko K. B. (1986). Multiple fates of newly synthesized neurofilament proteins: evidence for a stationary neurofilament network distributed nonuniformly along axons of retinal ganglion cell neurons. *J. Cell Biol.* 102, 647-659. 10.1083/jcb.102.2.647 2418034 PMC2114090

[BIO028795C49] Pant H. C. (1988). Dephosphorylation of neurofilament proteins enhances their susceptibility to degradation by calpain. *Biochem. J.* 256, 665-668. 10.1042/bj2560665 2851997 PMC1135461

[BIO028795C50] Pant H. C. and Veeranna (1995). Neurofilament phosphorylation. *Biochem. Cell Biol.* 73, 575-592. 10.1139/o95-063 8714676

[BIO028795C51] Perng M. D., Cairns L., van den IJssel P., Prescott A., Hutcheson A. M. and Quinlan R. A. (1999). Intermediate filament interactions can be altered by HSP27 and alphaB-crystallin. *J. Cell Sci.* 112, 2099-2112. 10362540 10.1242/jcs.112.13.2099

[BIO028795C52] Qin J. Y., Zhang L., Clift K. L., Hulur I., Xiang A. P., Ren B.-Z. and Lahn B. T. (2010). Systematic comparison of constitutive promoters and the doxycycline-inducible promoter. *PLoS ONE* 5, e10611 10.1371/journal.pone.0010611 20485554 PMC2868906

[BIO028795C53] Rao M. V. and Nixon R. A. (2003). Defective neurofilament transport in mouse models of amyotrophic lateral sclerosis: a review. *Neurochem. Res.* 28, 1041-1047. 10.1023/A:1023259207015 12737529

[BIO028795C54] Rao M. V., Yuan A., Campbell J., Kumar A. and Nixon R. A. (2012). The C-terminal domains of NF-H and NF-M subunits maintain axonal neurofilament content by blocking turnover of the stationary neurofilament network. *PLoS ONE* 7, e44320 10.1371/journal.pone.0044320 23028520 PMC3448626

[BIO028795C55] Ratnam J. and Teichberg V. I. (2005). Neurofilament-light increases the cell surface expression of the N-methyl-D-aspartate receptor and prevents its ubiquitination. *J. Neurochem.* 92, 878-885. 10.1111/j.1471-4159.2004.02936.x 15686490

[BIO028795C56] Roy S., Coffee P., Smith G., Liem R. K., Brady S. T. and Black M. M. (2000). Neurofilaments are transported rapidly but intermittently in axons: implications for slow axonal transport. *J. Neurosci.* 20, 6849-6861. 10995829 10.1523/JNEUROSCI.20-18-06849.2000 PMC6772820

[BIO028795C57] Shah J. V., Flanagan L. A., Janmey P. A. and Leterrier J.-F. (2000). Bidirectional translocation of neurofilaments along microtubules mediated in part by dynein/dynactin. *Mol. Biol. Cell* 11, 3495-3508. 10.1091/mbc.11.10.3495 11029051 PMC15009

[BIO028795C58] Shea T. B. and Beermann M. L. (1994). Respective roles of neurofilaments, microtubules, MAP1B, and tau in neurite outgrowth and stabilization. *Mol. Biol. Cell* 5, 863-875. 10.1091/mbc.5.8.863 7803854 PMC301107

[BIO028795C59] Shea T. B. and Flanagan L. A. (2001). Kinesin, dynein and neurofilament transport. *Trends Neurosci.* 24, 644-648. 10.1016/S0166-2236(00)01919-6 11672808

[BIO028795C60] Shea T. B. and Lee S. (2011). Neurofilament phosphorylation regulates axonal transport by an indirect mechanism: a merging of opposing hypotheses. *Cytoskeleton* 68, 589-595. 10.1002/cm.20535 21990272

[BIO028795C61] Shea T. B. and Yabe J. (2000). Occam's razor slices through the mysteries of neurofilament axonal transport: can it really be so simple? *Traffic* 1, 522-523. 10.1034/j.1600-0854.2000.010610.x 11208138

[BIO028795C62] Shea T. B., Sihag R. K. and Nixon R. A. (1990). Dynamics of phosphorylation and assembly of the high molecular weight neurofilament subunit in NB2a/d1 neuroblastoma. *J. Neurochem.* 55, 1784-1792. 10.1111/j.1471-4159.1990.tb04969.x 2213024

[BIO028795C63] Shea T. B., Paskevich P. A. and Beermann M. L. (1993). The protein phosphatase inhibitor okadaic acid increases axonal neurofilaments and neurite caliber, and decreases axonal microtubules in NB2a/d1 cells. *J. Neurosci. Res.* 35, 507-521. 10.1002/jnr.490350507 8397305

[BIO028795C64] Shea T. B., Lee S., Kushkuhley J. and Chan W.-K. H. (2009). Neurofilament dynamics: a tug of war by microtubule motors. *Future Neurol.* 4, 351-362. 10.2217/fnl.09.4

[BIO028795C65] Sunil N., Lee S. and Shea T. B. (2012). Interference with kinesin-based anterograde neurofilament axonal transport increases neurofilament-neurofilament bundling. *Cytoskeleton* 69, 371-379. 10.1002/cm.21030 22434685

[BIO028795C66] Szebenyi G., Smith G. M., Li P. and Brady S. T. (2002). Overexpression of neurofilament H disrupts normal cell structure and function. *J. Neurosci. Res.* 68, 185-198. 10.1002/jnr.10212 11948664

[BIO028795C67] Tokuyasu K. T., Dutton A. H. and Singer S. J. (1983). Immunoelectron microscopic studies of desmin (skeletin) localization and intermediate filament organization in chicken skeletal muscle. *J. Cell Biol.* 96, 1727-1735. 10.1083/jcb.96.6.1727 6343403 PMC2112467

[BIO028795C68] Trivedi N., Jung P. and Brown A. (2007). Neurofilaments switch between distinct mobile and stationary states during their transport along axons. *J. Neurosci.* 27, 507-516. 10.1523/JNEUROSCI.4227-06.2007 17234583 PMC1933499

[BIO028795C69] Uchida A., Alami N. H. and Brown A. (2009). Tight functional coupling of kinesin-1A and dynein motors in the bidirectional transport of neurofilaments. *Mol. Biol. Cell.* 20, 4997-5006. 10.1091/mbc.E09-04-0304 19812246 PMC2785742

[BIO028795C71] Vohnoutka R. B., Boumil E. F., Liu Y., Uchida A., Pant H. C. and Shea T. B. (2017). Influence of a GSK3β phosphorylation site within the proximal C-terminus of Neurofilament-H on neurofilament dynamics. *Biol. Open* 6, 1516-1527. 10.1242/bio.028522 28882840 PMC5665472

[BIO028795C72] Wagner O. I., Rammensee S., Korde N., Wen Q., Leterrier J.-F. and Janmey P. A. (2007). Softness, strength and self-repair in intermediate filament networks. *Exp. Cell Res.* 313, 2228-2235. 10.1016/j.yexcr.2007.04.025 17524395 PMC2709732

[BIO028795C73] Wang L. and Brown A. (2001). Rapid intermittent movement of axonal neurofilaments observed by fluorescence photobleaching. *Mol. Biol. Cell* 12, 3257-3267. 10.1091/mbc.12.10.3257 11598207 PMC60171

[BIO028795C74] Wang L., Ho C.-L., Sun D., Liem R. K. H. and Brown A. (2000). Rapid movement of axonal neurofilaments interrupted by prolonged pauses. *Nat. Cell Biol.* 2, 137-141. 10.1038/35004008 10707083

[BIO028795C75] Whitlon D. S. and Baas P. W. (1992). Improved methods for using glass coverslips in cell culture and electron microscopy. *J. Histochem. Cytochem.* 40, 875-877. 10.1177/40.6.1588032 1588032

[BIO028795C78] Yabe J. T., Pimenta A. and Shea T. B. (1999). Kinesin-mediated transport of neurofilament protein oligomers in growing axons. *J. Cell Sci.* 112, 3799-3814. 10523515 10.1242/jcs.112.21.3799

[BIO028795C79] Yabe J. T., Jung C., Chan W. K.-H. and Shea T. B. (2000). Phospho-dependent association of neurofilament proteins with kinesin in situ. *Cell Motil. Cytoskeleton* 42, 230-240. 10.1002/(SICI)1097-0169(200004)45:4<249::AID-CM1>3.0.CO;2-M 10744858

[BIO028795C80] Yabe J. T., Chylinski T., Wang F. S., Pimenta A., Kattar S. D., Linsley M. D., Chan W. K. and Shea T. B. (2001a). Neurofilaments consist of distinct populations that can be distinguished by C-terminal phosphorylation, bundling, and axonal transport rate in growing axonal neurites. *J. Neurosci.* 21, 2195-2205. 11264295 10.1523/JNEUROSCI.21-07-02195.2001 PMC6762414

[BIO028795C81] Yabe J. T., Wang F.-S., Chylinski T., Katchmar T. and Shea T. B. (2001b). Selective accumulation of the high molecular weight neurofilament subunit within the distal region of growing axonal neurites. *Cell Motil. Cytoskeleton* 50, 1-12. 10.1002/cm.1037 11746668

[BIO028795C82] Yuan A., Rao M. V., Sasaki T., Chen Y., Kumar A., Veeranna, Liem R. K. H., Eyer J., Peterson A. C., Julien J.-P. et al. (2006a). Alpha-internexin is structurally and functionally associated with the neurofilament triplet proteins in the mature CNS. *J. Neurosci.* 26, 10006-10019. 10.1523/JNEUROSCI.2580-06.2006 17005864 PMC6674481

[BIO028795C83] Yuan A., Nixon R. A. and Rao M. V. (2006b). Deleting the phosphorylated tail domain of the neurofilament heavy subunit does not alter neurofilament transport rate in vivo. *Neurosci. Lett.* 393, 264-268. 10.1016/j.neulet.2005.10.029 16266786

[BIO028795C84] Yuan A., Sasaki T., Rao M. V., Kumar A., Kanumuri V., Dunlop D. S., Liem R. K. and Nixon R. A. (2009). Neurofilaments form a highly stable stationary cytoskeleton after reaching a critical level in axons. *J. Neurosci.* 29, 11316-11329. 10.1523/JNEUROSCI.1942-09.2009 19741138 PMC2788791

[BIO028795C85] Yuan A., Sasaki T., Kumar A., Peterhoff C. M., Rao M. V., Liem R. K., Julien J.-P. and Nixon R. A. (2012). Peripherin is a subunit of peripheral nerve neurofilaments: implications for differential vulnerability of CNS and peripheral nervous system axons. *J. Neurosci.* 32, 8501-8508. 10.1523/JNEUROSCI.1081-12.2012 22723690 PMC3405552

[BIO028795C86] Yuan A., Hassinger L., Rao M. V., Julien J.-P., Miller C. C. J. and Nixon R. A. (2015). Dissociation of axonal neurofilament content from its transport rate. *PLoS ONE* 10, e0133848 10.1371/journal.pone.0133848 26208164 PMC4514674

[BIO028795C87] Yuan A., Rao M. V., Veeranna, and Nixon R. A. (2017). Neurofilaments and neurofilament proteins in health and disease. *Cold Spring Harb. Perspect. Biol.* 9, a018309 10.1101/cshperspect.a018309 28373358 PMC5378049

